# LncRNA TRPM2-AS promotes endometrial carcinoma progression and angiogenesis via targeting miR-497-5p/SPP1 axis

**DOI:** 10.1186/s11658-024-00612-7

**Published:** 2024-07-02

**Authors:** Hanbo Ma, Fengyun Weng, Xiaowen Tong, Huaifang Li, Yinan Yao, Jiangjing Yuan

**Affiliations:** 1 Department of Obstetrics and Gynecology, International Peace Maternity and Child Health Hospital, School of Medicine, Shanghai Jiao Tong University, Shanghai, 200030 China; 2 Shanghai Key Laboratory of Embryo Original Diseases, Shanghai, 200030 China; 3 Department of Obstetrics and Gynecology, Tongji Hospital of Tongji University, Tongji University School of Medicine, Shanghai, 200065 China; 4 Department of Obstetrics and Gynecology, Shangyu People’s Hospital of Shaoxing, Zhejiang, 312300 China

**Keywords:** LncRNA TRPM2-AS, Endometrial carcinoma, Angiogenesis, MiR-497-5p, SPP1

## Abstract

**Background:**

Anti-angiogenic therapy has become one of the effective treatment methods for tumors. Long noncoding RNAs (lncRNAs) are emerging as important regulators of tumorigenesis and angiogenesis in EC. However, the underlying mechanisms of lncRNA TRPM2-AS in EC are still not clear.

**Methods:**

We screened the differently expressed lncRNAs that were highly associated with poor prognosis and angiogenesis of EC by bioinformatics analysis, and constructed a ceRNA network based on the prognostic lncRNAs. The subcellular localization of TRPM2-AS was determined by fluorescence in situ hybridization (FISH) and nuclear cytoplasmic fractionation assay. CCK-8, EdU, transwell, western blot, qRT-PCR and endothelial tube formation assay were used to evaluate the effects of TRPM2-AS on the proliferation, invasion, migration of EC cells and angiogenesis. The targeted microRNA (miRNA) of TRPM2-AS was predicted by bioinformatic methods. The interaction between TRPM2-AS and miR497-5p, miR497-5p and SPP1 were analyzed by RNA immunoprecipitation and dual-luciferase reporter assay. A subcutaneous tumor model was used to explore TRPM2-AS’s function in vivo. CIBERSORT was used to analyze the correlation between TRPM2-AS and immune cell immersion in EC.

**Results:**

We found that the expression of TRPM2-AS and SPP1 was aberrantly upregulated, while miR-497-5p expression was significantly downregulated in EC tissues and cells. TRPM2-AS was closely correlated with the angiogenesis and poor prognosis in EC patients. Mechanistically, TRPM2-AS could sponge miR-497-5p to release SPP1, thus promoting the proliferation, invasion and migration of EC cells and angiogenesis of HUVECs. Knockdown of TRPM2-AS in xenograft mouse model inhibited tumor proliferation and angiogenesis in vivo. In addition, TRPM2-AS plays a vital role in regulating the tumor immune microenvironment of EC, overexpression of TRPM2-AS in EC cells stimulated the polarization of M2 macrophages and angiogenesis through secreting SPP1 enriched exosomes.

**Conclusion:**

The depletion of TRPM2-AS inhibits the oncogenicity of EC by targeting the miR-497-5p/SPP1 axis. This study offers a better understanding of TRPM2-AS’s role in regulating angiogenesis and provides a novel target for EC treatment.

**Supplementary Information:**

The online version contains supplementary material available at 10.1186/s11658-024-00612-7.

## Introduction

Endometrial carcinoma (EC) is an epithelial malignant tumor and one of the most common gynecologic cancer. There will be approximately 66,200 new cases and 13,030 deaths in 2023 in America [1]. The incidence and disease-associated mortality of EC are increasing [2]. Although 67% of patients present with early-stage disease, which has an 81% 5-year overall survival (OS), the 5-year OS for stage IVA and IVB EC are only 17% and 15%, respectively [3]. Despite the emergency of novel strategies to treat patients with advanced EC, the efficacy is still limited. A better understanding of the underlying mechanism of the progression of EC is crucial for precision treatment.

Non-coding RNAs (ncRNAs) are widely involved in various biological and pathological processes, they can be divided into long non-coding RNAs (lncRNAs) and microRNAs (miRNAs) based on their size [4]. LncRNAs have limited protein-coding potential owing to the lacking open reading frames, but they play critical roles in transcriptional regulation, epigenetic regulation of chromatin modification, and post-transcriptional regulation [5]. Human lncRNA TRPM2-AS (TRPM2-AS) is an antisense RNA of TRPM2, which is located at chromosome 21 and 875 nucleotides in length [6]. Studies have shown that the expression of TRPM2-AS is closely associated with tumor progression. For instance, TRPM2-AS can enhance cisplatin resistance and promote ovarian cancer progression [7]. TRPM2-AS enhanced the progression of bladder cancer by downregulating miR-22-3p and thus increasing GINS2 expression [8]. However, the function of TRPM2-AS in EC and the underlying mechanism are still poorly understood.

Angiogenesis is a process that new blood vessels develop from pre-existing blood vessels upon the stimulation of angiogenic factors. The angiogenesis-related factors promote the proliferation and migration of endothelial cells by degrading the extravascular matrix and basement membrane, resulting in the rearrangement of endothelial cells to form new vascular network [9]. Angiogenesis is vital for embryo development, wound healing, menstrual cycling and other normal physiological processes [9]. It also plays important roles in pathologic processes, including endometriosis and malignancies [10]. Tumor cells can obtain nutrients and oxygen required for growth from neovascularization. Neoangiogenesis promotes tumor growth and inhibits cancer cell apoptosis, which incites the recurrence and metastasis of tumor. Anti-angiogenesis therapies have been widely used in various cancers, but they present mixed results in EC treatment [11]. Identification of new targets and pathways of angiogenesis can provide novel insights for EC treatment.

The tumor microenvironment (TME) is composed of cancer cells, immune cells, mesenchymal cells, endothelial cells and non-cellular components such as cytokines and chemotaxis [12]. A large number of studies have shown that TME plays an important role in tumorigenesis, metastasis, angiogenesis and drug resistance [13–16]. Therefore, targeted therapy of macrophages in TME is a critical anti-cancer strategy.

As an integrin-binding matricellular protein, secreted phosphoprotein 1 (SPP1, OPN) take parts in the regulation of multiple processes such as tumourigenesis, angiogenesis, cell-mediated immunity and metastasis [17, 18]. Studies also show that SPP1 is overexpressed in various malignant tumor [19], including breast cancer, gastric cancer, lung cancer [20–22].

In this study, we explored the role of TRPM2-AS/miR-497-5p/SPP1 in promoting angiogenesis and progression of EC. Besides, we revealed that EC cells-derived exosomes which contains TRPM2-AS could promote angiogenesis through regulating M2 macrophages polarization. Our findings elucidate the underlying mechanisms of TRPM2-AS in regulating angiogenesis of EC and provides novel targets for EC targeted therapy.

## Materials and methods

### Data collection

The clinical data and correlated mRNA expression of 543 EC and 35 normal cases were obtained from The Cancer Genome Atlas (TCGA). Moreover, 161 angiogenesis-related genes were retrieved from the MSigDB database.

### Screening of differentially expressed mRNAs, miRNAs, and lncRNAs between EC and non-cancer tissues

First, we obtained the raw counts of mRNAs, miRNAs, and lncRNAs expression profiles from TCGA. The RNA sequencing data of EC contains 19381 mRNA, 2226 miRNA, and 14042 lncRNA expression profiles. Then, the differentially expressed genes (DEGs) were calculated using the DESeq R package. The DEGs of the dataset with an absolute log2 fold change (FC) > 1 and an adjusted *P*-value of < 0.05 were considered for subsequent analysis.

### Construction of the differentially expressed and angiogenesis-related lncRNA prognostic model

Univariate, least absolute shrinkage and selection operator (LASSO), and multivariate Cox regression analyses were employed to investigate the correlation between patient overall survival (OS) and the expression level of each gene. The gene was considered significant when the *P*-value was < 0.05 in the univariate Cox regression analysis. Next, we applied a LASSO‐penalized Cox regression to further reduce the dimensionality of prognostic genes. Finally, multivariate Cox regression analysis was employed to select the best genes. A prognosis risk score system was established based on four lncRNAs which contained TRPM2-AS, HAND2-AS1, LINC00957 and AC011472.4. The patients were divided into low-risk and high-risk groups based on the risk score of the four lncRNAs. Kaplan‐Meier (K-M) survival curves for the cases with low or high risk were generated. Then, time‐dependent receiver operating characteristic (ROC) curve analysis was conducted and the area under the curve (AUC) for 3‐year, 5‐year, and 7‐year OS were calculated to determine the prediction accuracy of our model by using the ‘survival ROC’ package in R.

### Correlation between clinical characteristics and risk scores

To determine whether the risk scores correlated the clinical conditions, the clinical pathological characteristics of EC that obtained from the TCGA-EC dataset were analyzed. Patients were stratified into predefined categories (age ≤ 55 years or age > 55 years, BMI ≤ 30 or > 30, stage I–II or stage III–IV, grade 1–2 or 3, pregnancies < 2 or ≥ 2, and peri-, post-, pre- or unknown menopause). OS analysis was performed between each subgroup.

To analyze the predictive value of the prognostic model in clinical variables (including age, stage, radiotherapy and chemotherapy) for patients with EC, univariate and multivariate Cox regression analyses were conducted, with clinical characteristics as independent variables and the OS as the dependent variable. All reported *P* values were two-sided. The hazard ratio (HR) and 95% confidence intervals were calculated.

### Gene set enrichment analyses

To explore the potential molecular mechanisms underlying our constructed prognostic gene signature, Gene Set Enrichment Analyses (GSEA) was performed to find enriched terms that correlated with the Kyoto Encyclopedia of Genes and Genomes (KEGG) pathway in C2; in C5, a gene set that contains genes annotated by the same gene ontology (GO) term. *P* < 0.05 was considered statistically significant.

### Construction of lncRNA-miRNA-mRNA ceRNA network

The lncRNA-miRNA-mRNA ceRNA network was based on the theory that lncRNA can directly interact by invoking miRNA sponges to regulate mRNA activity. Then, the predicted miRNAs were intersected with DE miRNAs and selected for further analysis. Interaction between miRNA and lncRNA was predicted by Starbase. Cytoscape software (version 3.4.0) was utilized to visualize the regulatory network.

### Clinical samples and cell lines

38 pairs of EC and adjacent normal tissues were collected in the Shanghai Tongji hospital. The research was approved by the Ethical Committee of Shanghai Tongji hospital. Fresh tissues were immediately frozen in liquid nitrogen for RNA extraction or fixed in 4% PFA for IHC staining. Human EC cell lines KLE (Cat. No.: FH0304), AN3CA (Cat. No.: FH0302), Ishikawa (Cat. No.: FH0305), RL95-2 (Cat. No.: FH0303), HEC1-A (Cat. No.: FH0307) HEC1-B (Cat. No.: FH0306), primary endometrial epithelial cells (EEC, Cat. No.: FH-H068), human monocytic cell line (THP-1, Cat. No.: FH0112) and human umbilical vein endothelial cells (HUVECs, Cat. No.: FH1122) were purchased from Shanghai FuHeng Biological Co., LTD.

### Cell culture and transfection

EC cell lines EEC were routinely cultured in DMEM/F12 medium (for EEC, KLE and RL95-2 cell lines), EMEM medium (for AN3CA and HEC1-B cell lines), McCoy's 5a Medium (for HEC1-A cells) or MEM medium (for Ishikawa cells) respectively. The above mediums were supplied with 10% fetal bovine serum (FBS) and 1% *P*/S. Cells were incubated in a 37 ℃ humidified incubator containing 5% CO_2_. HUVECs were cultured in human large vessel endothelial cell basal medium supplemented with LSGS and *P*/S. THP-1 cells were cultured in RPMI 1640 medium (Sigma) supplemented with 10% FBS. To induce the differentiation of THP-1 cells into M0 macrophages, cell cultures were treated with 100 ng/ml phorbol 12-myristate-13acetate (PMA, Sigma). For M1 macrophage polarization, 20 ng/ml IFN-gamma and 10 pg/ml LPS were added into M0 macrophages. For M2 macrophage polarization, M0 macrophages were incubated with 20 ng/ml IL-4 and 20 ng/ml IL-13. To evaluate EC microenvironment’s effect on macrophage polarization, M0 macrophages were co-cultured with conditioned medium that collected from Ishikawa cells under different transfection treatment.

TRPM2-AS silencing and overexpression vectors were purchased from Shanghai Genechem Co., LTD. Short hair stranded RNA that targeting TRPM2-AS (sh-TRPM2-AS), TRPM2-AS overexpression vector (OE-TRPM2-AS) and the control vectors (sh-NC and OE-NC) were introduced into recipient cells via cell transfection using Lipofectamine™ 3000 48 h after the transfection, cell samples were collected for subsequent experiments.

The overexpression and knockdown lentivirus of TRPM2-AS were packaged at Genechem. Ishikawa cells were seeded in 6-well plates. After the cell confluence reached 50%, the lentivirus of sh-NC, shTRPM2-AS, vector or TRPM2-AS was added in the cells. For selection of stably transfected cells, fresh medium containing 2 µg/ml puromycin antibiotic was added 48 h after infection. Cells were changed with freshly prepared selective medium every 2 days until the untransfected Ishikawa cells has all been killed. The stably transfected cells lines were cultured in medium that contained 1 µg/ml puromycin, and qRT-PCR analysis was conducted to detect the expression of TRPM2-AS.

### CCK-8 assay

The transfected EC cells were seeded into 96-well plates at the density of 2 × 10^3^ per well. After cultured for 0, 24, 48 or 72 h, 10 μL CCK-8 detection solution (Beyotime) was added to each well, and the cells were incubated at 37 ℃ for 4 h before detection. The absorbance was measured by the Bio Tek microplate reader at 450 nm.

### EdU assay

The transfected EC cells were seeded into 24 well plates that contains round coverslip and cultured overnight. EdU solution (final working concentration at 10 μM) were added into each well to label proliferating cells. The cell cultures were incubated at 37 ℃ for 2 h. After the labeling of EdU, medium was removed, and the cells were incubated with 500 μL 4% paraformaldehyde at room temperature for 15 min. Then cells were incubated with PBS containing 0.3% Triton X-100 for 10 min at room temperature. After washed the cells for 3 times, 100 μL click reaction solution were added, and cells were incubated at room temperature for 30 min away from light. Cells were washed for 3 times and 200 μL Hoechst 33342 staining solution were added into each well, then incubated at room temperature away from light for 10 min. After the final wash, cell slides were mounted and photographed with confocal microscope (Zeiss, LSM880).

### Transwell assay

The migration and invasion of EC cells were detected by transwell assay. After 12 h of starvation, 200 μL 2 × 10^5^/mL cell suspension were added into each transwell chamber with 24 well plate. For invasion transwell assay, chambers were pretreated with matrigel. The complete culture medium was added into the lower chamber. After cultured for 48 h, cells were fixed with 4% paraformaldehyde for 15 min, and stained with crystal violet staining solution (Beyotime) for 10 min. Pictures were captured by microscope (Olympus, FV3000) and the average number of transmembrane cells were counted by Image J.

### Quantitative reverse transcription-polymerase chain reaction (qRT-PCR)

TRIzol reagent (Invitrogen) was used to isolate RNA from cells and tumor tissues, and TRIzol LS reagent (Invitrogen, Carlsbad, USA) was used to extract the RNA from exosome. During the precipitation step, glycogen was added to promote the precipitation of RNA.

RiboSCRIPTTM mRNA/lncRNA qRT-PCR Kit (C11030-2) was used to perform reverse transcription and qRT-PCR of lncRNAs. Mir-X miRNA First-Strand Synthesis and TB Green™ qRT-PCR Kit (TaKaRa) were used to determine the expression of miRNAs. PrimeScript RT reagent and TB Green Premix Ex TaqII kit were used to analysis the expression of SPP1, E-cadherin, N-cadherin, Vimentin, CD31, CD80, CD86, iNOS, CD163 and CD206.

The primers for miR-497-5p, miR-15b-5p, miR-132-5p, miR-138-5p, miR-942-5p, U6, lncRNA TRPM2-AS, HAND2-AS1, LINC00957, AC011472.4 and GAPDH were obtained from RiboBio. Primers of SPP1, E-cadherin, N-cadherin, Vimentin, CD31, CD80, CD86, iNOS, CD163 and CD206 were synthesized by Sangon Biotech (Shanghai, China). qRT-PCR was performed on QuantStudio Q5 system and the data were analyzed by 2-ΔΔCt method.

### Western blotting

Cells or tissues were lysed with RIPA buffer (Beyotime) supplied with PMSF. The lysates were incubated on ice for 30 min and centrifuge at 4 ℃ 12,000 rpm for 15 min. The protein concentration was determined by BCA protein assay kit. Supernatant of the lysate was transferred into clean tubes, and the samples were boiled with 5 × SDS-PAGE loading buffer at 95 ℃ for 5 min. The same amount of protein (20 μg) were loaded into the wells of SDS-PAGE gel, and the gels were run at a constant voltage of 150 V for 65 min. Then the protein was transferred from the gel to the PVDF membrane at a constant current of 350 mA. The membranes were blocked in 5% non-fat milk in TBST at room temperature for 1 h and incubated with primary antibody at 4 ℃ overnight.

The primary antibodies used were as follows: E-cadherin (abd40772, 1:10,000), N-cadherin (ab76011, 1:5000), Vimentin (ab92547, 1:1000), SPP1 (ab283669, 1:1000), CD31 (ab281583, 1:1000), CD63(ab134045, 1:5000), CD81 (ab109201, 1:5000), TSG101 (ab125011, 1:5000), and GAPDH (ab8245, 1:10,000; Abcam, Cambridge, UK). After washed with TBST for 3 times, the membranes were incubated with secondary antibodies respectively at room temperature for 1 h. The enhanced chemiluminescence (ECL) system was used to visualize the protein bands, and the intensity of bands were measured by Image J.

### RNA immunoprecipitation (RIP)

Cells were lysed in RIPA buffer supplied with proteinase inhibitor on ice for 30 min. After centrifuge at 13,000 rpm 4 °C for 15 min, the supernatant was collected and transferred into RNase free tube. Magnetic beads were washed by RIPA buffer and incubated with Ago2 antibody at room temperature for 30 min. Equal amount of IgG was added for the control group. After coupling the antibody to the magnetic beads, 500 ul RIPA buffer was added to remove the unbound antibodies. The beads were resuspended in 900 ul RIPA buffer and 100 ul cell lysate were added, the mixture was rotated at 4 °C overnight. After washed with RIPA buffer, the beads were incubated with proteinase K to remove proteins. The RNA in the immunoprecipitation complex were purified and detected by qRT-PCR. 

### Dual-luciferase reporter assay

The wild-type and mutant sequence of the binding site between TRPM2-AS and miR-497-5p were cloned into the vector of dual-luciferase reporter system. The reporter vector and miR-497-5p mimic of TRPM2-AS were transfected into 293 T cells. After 48 h, cells were collected in lysis buffer and underwent subsequent procedures according to the manufacturer’s instructions (Dual-Luciferase™ ReporterAssay Systems, Promega™ E1910).

### Exosome purification

When the confluence of the Ishikawa cells reached 70 to 80%, serum-free medium (Gibco) were changed. After cultured for 48 h, the supernatant was collected and centrifuged stepwise at 300×*g* for 10 min, 2000×*g* for 10 min, and 10,000×*g* for 10 min 4 ℃ to remove the cell debris. The supernatant was centrifuged at 100,000×*g* for 70 min twice and the pellet was resuspended in PBS.

### Xenograft model

Animal experiments were approved by the Laboratory Animal Welfare Ethics Committee of Shanghai Tongji Hospital. BALB/c female nude mice (*n* = 24) aged 4–6 weeks were purchased from Shanghai JieSiJie Laboratory Animal Co., Ltd. Ishikawa cells (1 × 10^6^) that stably expressed the sh-TRPM2-AS, sh-NC, TRPM2-AS-OE or Vector were subcutaneously injected into the BALB/c female nude mice (4–6 weeks old). The volume of tumor was measured every 4 days, and the nude mice were sacrificed after 4 weeks. The tumor was exfoliated, photographed and weighted for further analysis.

### Statistical analysis

Graphs and statistical analyses were performed with Graph Pad Prism 7. Student *t*-test was used between two groups, and one-way ANOVA followed by Tukey’s post hoc test was utilized for comparing more than two groups. Data are presented as mean ± SEM, *P* < 0.05 was considered statistically significant.

## Results

### Identification of differentially expressed and angiogenesis-related lncRNAs prognostic signature

**Fig. 1:**
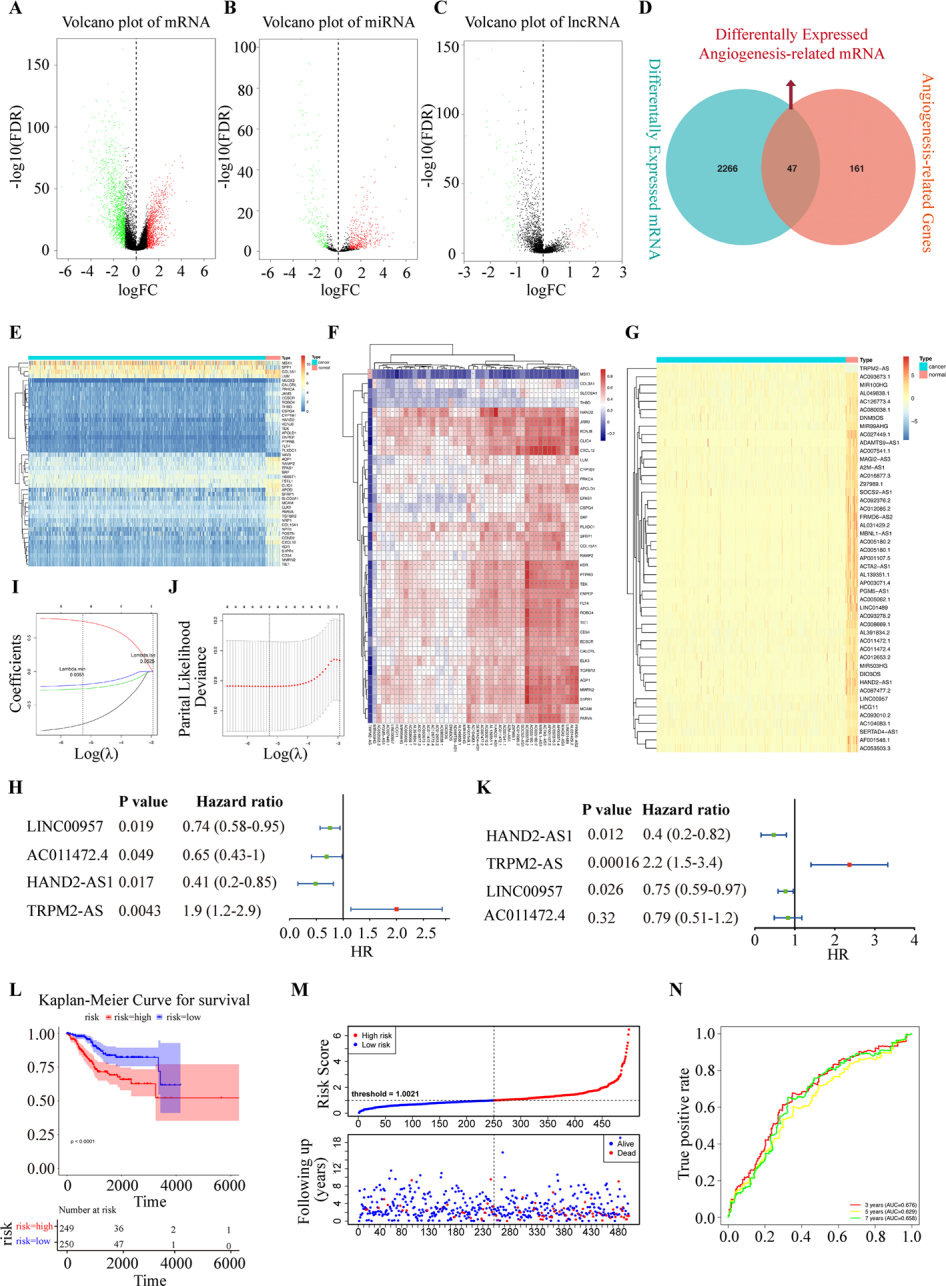
Identification of differentially expressed angiogenesis-related and prognostic lncRNAs in EC. **A** Volcano plot analysis of differentially expressed mRNAs in EC. **B** Volcano plot analysis of differentially expressed miRNAs in EC. **C** Volcano plot of differentially expressed lncRNAs in EC. **D** Venn diagram of differentially expressed mRNAs at the intersection of angiogenesis-related genes. **E** Expression profiles of 47 differentially expressed angiogenesis-related mRNAs in EC and normal tissues. **F** Correlation between 47 differentially expressed lncRNAs and angiogenesis-related genes. **G** Expression profiles of 47 differentially expressed angiogenesis-related lncRNAs in EC and normal tissues. **H** Identification of four lncRNAs that correlated with OS of EC by univariate Cox regression analysis. **I** LASSO coefficient profiles of the 4 prognostics-related lncRNAs. **J** The tuning parameter (*λ*) in the LASSO model were determined by tenfold cross-validation. **K** The multivariate cox regression analysis of four lncRNAs. **L** Kaplan–Meier overall survival curve of high-risk group and low-risk group. **M** The risk score distribution and sample survival time of high-risk group and low-risk group. **N** ROC curves of 3-, 5- and 7-year OS

To identify the differentially expressed genes in EC, we analyzed the expression profiles of mRNA, miRNA and lncRNA in the uterine corpus endometrial carcinoma (UCEC) of TCGA database. There are 2266 mRNAs, 656 miRNAs, and 119 lncRNAs that were differentially expressed in EC. We highlighted the up-regulated genes by red and down-regulated genes by green in volcano plots. 992 mRNAs were up-regulated and 1274 mRNAs were down-regulated (Fig. 1A). 445 miRNAs were significantly increased and 211 miRNAs were decreased in EC tissues compared with control (Fig. 1B). Additionally, 119 lncRNAs were differentially expressed in EC samples, among which 40 lncRNAs were up-regulated and 79 lncRNAs were down-regulated (Fig. 1C). To screen key genes that regulating angiogenesis in EC, we take intersection of 2266 differentially expressed genes in EC and 161 angiogenesis-related genes that downloaded from “MSigDB” database. There are 47 differentially expressed and angiogenesis-related (DE-AR) mRNAs were obtained, among which 4 genes (HS6ST1, MSX1, VAV3, SPP1) were up-regulated and 43 genes were down-regulated, with the thresholds of |log_2_ FC|> 1 and *P* < 0.05 (Fig. 1D, E).

To find out the lncRNA that play crucial roles in regulating angiogenesis of EC, we conducted Pearson correlation analysis between 119 DE lncRNAs and 47 DE-AR mRNAs. The lncRNA was defined as DE-AR lncRNA when its expression level correlates with that of the DE-AR mRNA (|Pearson *R*|> 0.3 and *P* < 0.05). 47 DE-AR lncRNAs were correlated with 37 DE-AR mRNAs in EC (Fig. 1F). Then, we analyzed the expression of 47 DE-AR lncRNAs in EC and normal tissues. The heatmap indicates that the expression of TRPM2-AS was increased in EC tissues compared with normal tissues, while the other 46 DE-AR lncRNAs were decreased in EC tissues (Fig. 1G).

To investigate the correlation between the DE-AR lncRNAs and overall survival (OS) of EC patients, we conducted a univariate Cox regression and identified 4 DE-AR lncRNAs that were significantly correlated with OS of EC patients. TRPM2-AS are associated with poor survival whereas HAND2-AS1, LINC00957, and AC011472.4 have negative coefficients (Fig. 1H). Least absolute shrinkage and selection operator (LASSO) regression analysis was performed, and cross validation was conducted. The results showed that when the variable is 4. The root mean square error of the model is the smallest and the corresponding *λ* value is 0.0051 (Fig. 1I–J). Finally, a multivariate Cox regression analysis was conducted, and 4 lncRNAs (TRPM2-AS, HAND2-AS1, LINC00957 and AC011472.4) were selected to build the predictive model (Fig. 1K). According to this signature, patients were divided into low-risk and high‐risk groups. The survival analysis shows that patients with low-risk score had a higher survival rate than patients with high-risk score (*P* < 0.0001, Fig. 1L, M). Time‐dependent ROC analysis shows that the AUC of the DE-AR lncRNAs signature was 0.676 at 3 years, 0.629 at 5 years, and 0.658 at 7 years, which indicates that the forecast model has high sensitivity and specificity (Fig. 1N).

Next, we explored the correlation between clinicopathological characteristics and prognostic signature. Clinicopathological features, which includes age, BMI, grade, menopause, pregnancies and stage, were collected from the TCGA‐EC dataset. As shown in Fig. S1A, part of the clinical factors such as age, grade and stage show significantly difference between low-risk and high-risk group. Besides, the expression of TRPM2-AS, AC011472.4, LINC00957 and HAND2-AS1 were also different in the above two groups.

Univariate and multivariate Cox regression analyses were conducted to evaluate the correlation between clinicopathological features and OS. Univariate Cox regression analysis shows that age, stage, grade and risk score were significantly correlated with OS of EC patient (Fig. S1B). Besides, the multivariate Cox regression analysis show that that age, stage, grade and risk score were independent prognostic factors (Fig. S1C). Moreover, we constructed the predictive nomogram to predict 1-, 2- and 3-year survival of EC patients (Fig. S1D).

To explore the interaction of the prognostic lncRNAs, DE miRNAs, and DE-AR mRNAs in EC, we built the ceRNA network. Among which, 8 miRNAs, 4 mRNAs and 2 lncRNAs were identified (Fig. S2A). It has been reported that HAND2-AS1 inhibits invasion and metastasis through activating neuromedin U in endometrioid endometrial carcinoma [23]. However, the role of TRPM2-AS in EC is still unknown. Therefore, we chose TRPM2-AS as candidate gene for further research.

### Increased expression of TRPM2-AS in EC correlates with poor prognosis

**Fig. 2:**
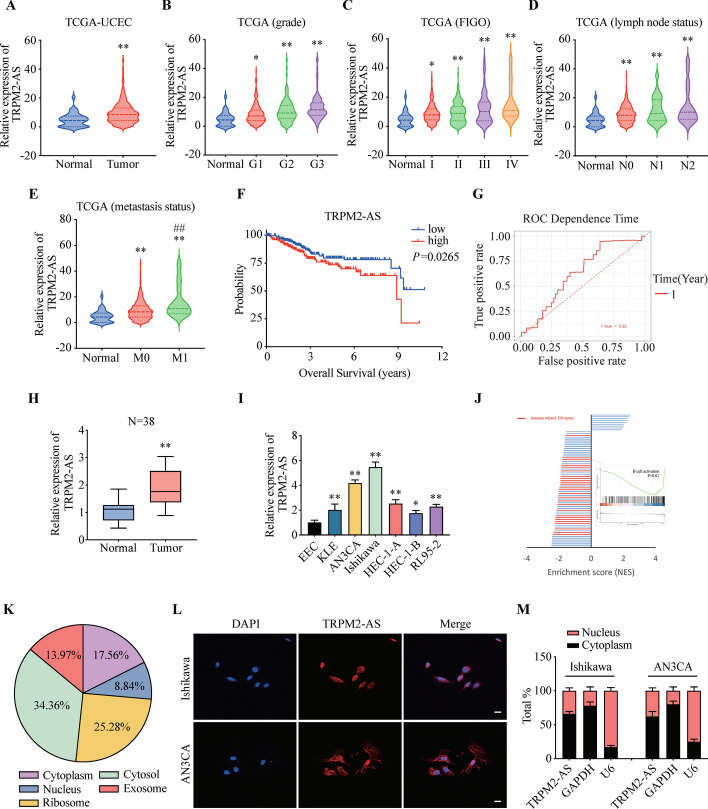
The expression of TRPM2-AS and its correlation with clinicopathologic features in EC. **A** The expression of TRPM2-AS in EC and normal tissues. **B**–**E** The expression patterns of TRPM2-AS based on grade (**B**), FIGO stage (**C**), N stage (**D**) and M stage (**E**) in TCGA UCEC dataset. **F** Kaplan–Meier curves of OS according to the TRPM2-AS’ expression in EC tissues. **G** 1-year ROC curve of OS showed the diagnostic value of TRPM2-AS in samples from TCGA UCEC dataset. **H** The expression of TRPM2-AS in EC and adjacent normal tissues. **I** The expression of TRPM2-AS in EC cell lines and ECC. **J** The gene enrichment analysis of TRPM2-AS in EC. **K** Subcellular localization of TRPM2-AS based on information from lncLocator database. **L** Fluorescence in situ hybridization assay showed the subcellular localization of TRPM2-AS in Ishikawa and AN3CA cells. **M** The distribution of TRPM2-AS in nucleus and cytoplasm of Ishikawa and AN3CA cells. Data were representative of three independent experiments and values were expressed in mean ± SD. (One-way ANOVA or Student’s *t*-test; *P < 0.05, **P < 0.01 as compared with normal)

To identify the expression of TRPM2-AS and potential roles in EC, we explored TCGA database and found that the expression of TRPM2-AS was increased in EC compared with normal tissues (Fig. 2A). Then, we analyzed the correlation between TRPM2-AS and clinicopathologic characteristics of EC. As shown in Fig. 2B, the expression of TRPM2-AS was higher in G1-G3 grade than normal tissues. Meanwhile, high expression of TRPM2-AS was also associated with advanced FIGO stage (Fig. 2C), lymphatic metastasis (Fig. 2D) and distant metastasis (Fig. 2E). These results indicated that the expression level of TRPM2-AS was positively correlated with advanced histological grade, FIGO stage, lymph node and distant metastasis. To investigate the prognostic values of TRPM2-AS, we analyzed the survival rate of low and high TRPM2-AS expression groups. The results showed that the overall survival rate of the high expression group was shorter than the low expression group (Fig. 2F). To evaluate the diagnostic value of TRPM2-AS, we conducted ROC curve analysis. The 1-year AUC value is 0.63, which indicates TRPM2-AS is a potential biomarker for EC (Fig. 2G). We assessed the expression of TRPM2-AS in 38 pairs of EC and adjacent normal tissues by qRT-PCR. Clinical information of these EC patients was presented in Table 1. TRPM2-AS was highly expressed in EC compared to adjacent tissues (Fig. 2H). Meanwhile, TRPM2-AS was also highly expressed in EC cell lines (KLE, AN3CA, Ishikawa, HEC-1A, HEC-1B and RL95-2) compared to EEC. Since the expression of TRPM2-AS was relatively high in Ishikawa and AN3CA cells, we selected them for subsequent experiments (Fig. 2I).

**Table 1:** Clinical information of EC patients (*n* = 38)

Pathological characteristics	No. of cases
Age (years)	
≤ 55	16
> 55	22
FIGO stage	
I–II	28
III–IV	10
Myometrium invasion	
≤ 1/2	23
> 1/2	15
Pathological type	
Endometrioid	27
Non-endometrioid	11
Histological grade	
Grade 1	20
Grade 2	13
Grade 3	5
Lymph node metastasis	
Positive	8
Negative	30

To explore the TRPM2-AS related signaling pathway, we performed gene set enrichment analysis (GSEA). GSEA revealed that immune signaling pathways were significant negatively enriched in TRPM2-AS highly expressed samples, which includes B cell activation and adaptive immune response signaling (Fig. 2J). The result suggested that TRPM2-AS plays a role in regulating microenvironment of immune cells in EC.

To predict the subcellular localization of TRPM2-AS, we analyzed the lncLocator database. The results showed that TRPM2-AS was mainly localized in the cytosol, ribosome and cytoplasm (Fig. 2K). Subsequently, we used nuclear and cytoplasmic extraction and in situ hybridization to examine the subcellular localization of TRPM2-AS in Ishikawa and AN3CA cells. TRPM2-AS was mainly localized in the cytoplasm (Fig. 2L, M), which suggested that TRPM2-AS might exert its biological function through the ceRNA mechanism.

### TRPM2-AS promotes EC progression and angiogenesis

**Fig. 3:**
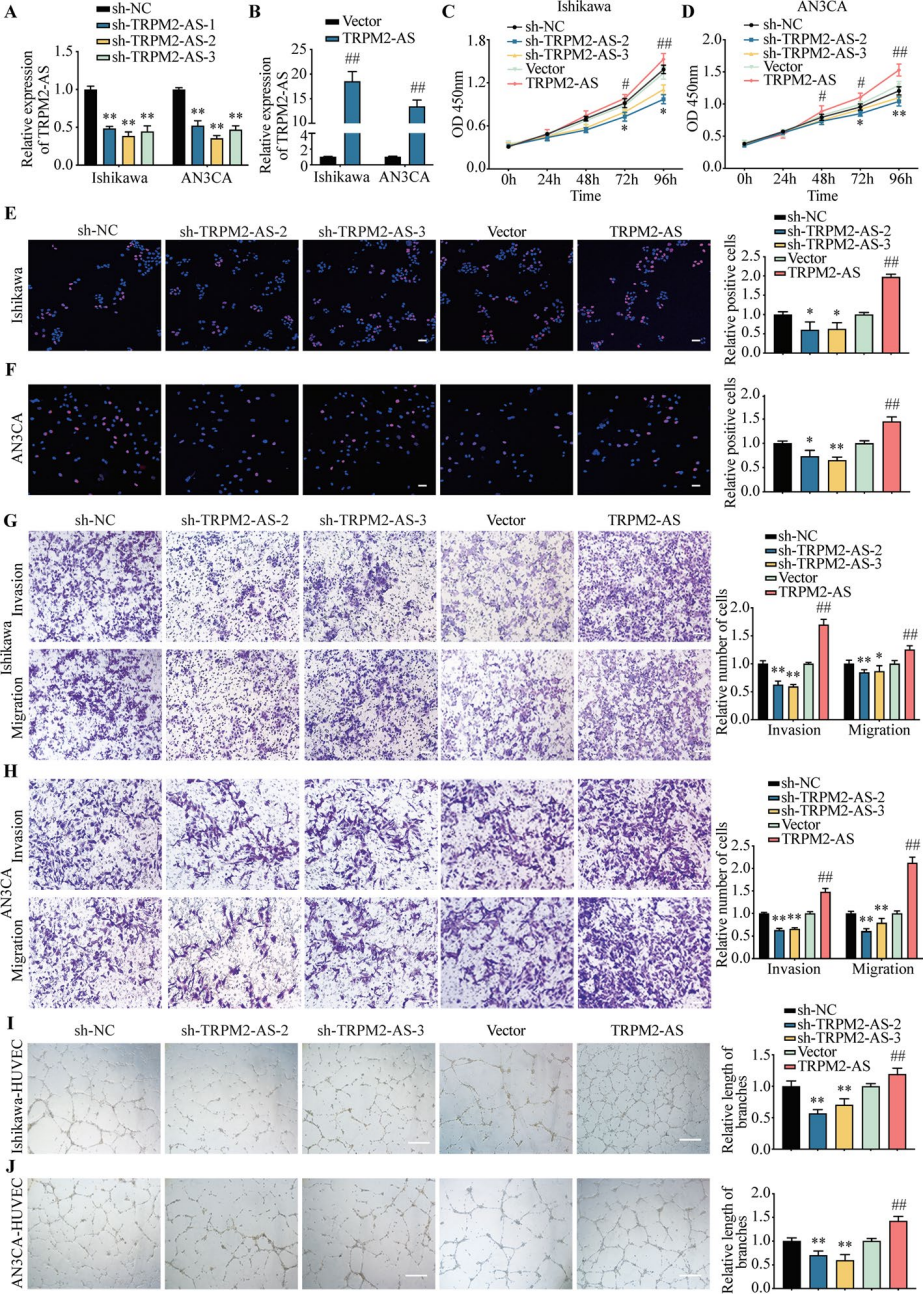
Effects of TRPM2-AS on the proliferation, migration, invasion of EC cells and angiogenesis of HUVECs. **A**, **B** The expression of TRPM2-AS in knockdown (**A**) or overexpression (**B**) groups by qRT-PCR. **C**, **D** CCK-8 assay detected the effects of TRPM2-AS on proliferation of Ishikawa (**C**) or AN3CA cells (**D**). **E**, **F** The EdU assay showed TRPM2-AS’s role in regulating the proliferation of Ishikawa (**E**) or AN3CA cells (**F**). **G**, **H** Transwell assay showed the invasion and migration ability of sh-TRPM2-AS or TRPM2-AS transfected Ishikawa (**G**) or AN3CA cells (**H**). **I**, **J** HUVEC tube formation assay revealed the effects of TRPM2-AS on angiogenesis. Data were representative of three independent experiments and values were expressed in mean ± SD. (One-way ANOVA or Student’s *t*-test; *P < 0.05, **P < 0.01 as compared with sh-NC; # *P* < 0.05, ## *P* < 0.01 as compared with vector)

To explore the function of TRPM2-AS in EC, we constructed the TRPM2-AS knockdown and overexpression plasmids. The knockdown efficiency of sh-TRPM2-AS in Ishikawa and AN3CA cells were tested. The qRT-PCR results showed that sh-TRPM2-AS-1, sh-TRPM2-AS-2 and sh-TRPM2-AS-3 could successfully knock down TRPM2-AS. sh-TRPM2-AS-2 and sh-TRPM2-AS-3 show stronger knockdown effect. So, we chose them for subsequent experiments (Fig. 3A). The efficiency of TRPM2-AS overexpression vectors in Ishikawa and AN3CA cells also has been tested by qRT-PCR (Fig. 3B).

Next, we investigated the role of TRPM2-AS in the proliferation, invasion, migration, angiogenesis and epithelial-mesenchymal transformation (EMT). CCK-8 assay and EdU staining showed that knockdown of TRPM2-AS inhibited the proliferation of Ishikawa and AN3CA cells compared with the control group. Conversely, overexpression of TRPM2-AS promoted the proliferation (Fig. 3C–F).

The transwell cell invasion assay showed that knockdown of TRPM2-AS inhibited the invasion of Ishikawa and AN3CA cells. On the contrary, overexpression of TRPM2-AS have the opposite effects. Transwell migration assays were also conducted and the results revealed that the number of cells crossing the chamber was reduced in TRPM2-AS knockdown group compared with the sh-NC group, which indicated that the migration of Ishikawa and AN3CA cells was inhibited by knockdown of TRPM2-AS. Overexpression of TRPM2-AS promoted the migration of EC cells (Fig. 3G, H). Our data demonstrated that TRPM2-AS promoted the invasion and migration of Ishikawa and AN3CA cells.

To explore the role of TRPM2-AS in angiogenesis, we co-cultured the HUVECs with conditional medium of sh-TRPM2-AS or TRPM2-AS infected Ishikawa and AN3CA cells. Compared with the sh-NC group, the formation of blood vessels was significantly reduced in the sh-TRPM2-AS group. On the contrary, the formation of capillaries was obviously increased in the TRPM2-AS overexpression group compared with the control group (Fig. 3I, J). These results indicated that TRPM2-AS promoted angiogenesis of HUVECs, and knockdown of TRPM2-AS reversed this effect.

In the sh-TRPM2-AS group, the expression of E-cadherin was significantly increased, while N-cadherin and Vimentin were decreased. Meanwhile, the level of CD31 (angiogenesis marker) was also reduced in sh-TRPM2-AS group, and overexpression of TRPM2-AS had the opposite effects (Fig. S3A-D). Our results suggest that upregulated TRPM2-AS plays an important role in the metastasis and angiogenesis of EC cells.

### TRPM2-AS regulate EC cells through miR-497-5p

**Fig. 4:**
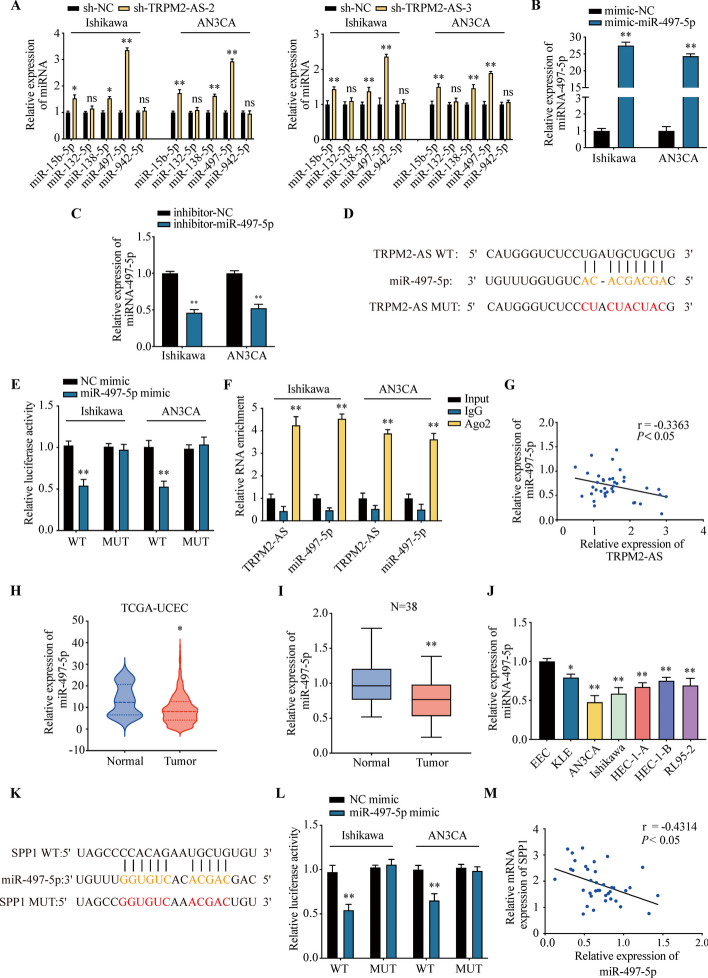
TRPM2-AS acts as a ceRNA to regulate SPP1 via sponging miR-497-5p. **A** The expression of miR-15b-5p, miR-132-5p, miR-138-5p, miR-497-5p and miR-942-5p in sh-TRPM2-AS-2 transfected EC cells were detected by qRT-PCR. **B** The expression of miR-497-5p in mimic-NC and miR-497-5p mimic groups were analyzed by qRT-PCR. **C** The knockdown effect of inhibitor-miR-497-5p was examined by qRT-PCR. **D** Predicted binding sites and mutant sequences between TRPM2-AS and miR-497-5p. **E** The luciferase activity was detected after co-transfection NC mimic or miR-497-5p mimic with dual-luciferase reporter plasmid for 48 h. **F** RNA immunoprecipitation assay showed the interaction between TRPM2-AS and miR-497-5p. **G** The expression of TRPM2-AS was negatively correlated with miR-497-5p. **H** Relative expression of miR-497-5p in TCGA-UCEC database. **I** The expression of miR-497-5p in EC tissues. **J** Relative expression of miR-497-5p in EC cell lines. **K** Binding sites and mutant sequences between miR-497-5p and SPP1. **L** The luciferase activity was detected after co-transfection NC mimic or miR-497-5p mimic with SPP1-WT or SPP1-MUT reporter plasmids. **M** The expression of miR-497-5p was negatively correlated with SPP1. Data were representative of three independent experiments and values were expressed in mean ± SD. (One-way ANOVA or Student’s *t*-test; *P < 0.05, **P < 0.01)

To screen the predicted downstream miRNAs of TRPM2-AS, we knocked down the TRPM2-AS in Ishikawa and AN3CA cells and detected the expression of miRNA by qRT-PCR. The results showed that the expression of miR-15-5p, miR-138-5p and miR-497-5p were increased after TRPM2-AS knockdown in these two cell lines. However, the expression of miR-132-5p and miR-942-5p did not show statistically significant difference between sh-TRPM2-AS and control group. Among them, the expression of miR-497-5p changed most obvious (Fig. 4A). Consequently, we chose it for further research.

The mimic and inhibitor of miR-497-5p were used to explore miR-497-5p’s role in Ishikawa and AN3CA cells. The expression of miR-497-5p was remarkably increased in miR-497-5p mimic group and decreased in miR-497-5p inhibitor group compared with control (Fig. 4B, C). The potential binding sites of TRPM2-AS and miR-497-5p were predicted by starbase (Fig. 4D). Luciferase assay showed that miR-497-5p decreased the activity of TRPM2-AS-WT in Ishikawa and AN3CA cells. However, the inhibitory effect was reversed by mutation of the binding site of TRPM2-AS and miR-497-5p (Fig. 4E). RNA immunoprecipitation assay also proved the interaction between TRPM2-AS and miR-497-5p (Fig. 4F). To further investigate the correlation between TRPM2-AS and miR-497-5p in clinical samples, we conducted the Pearson curve analysis and the results showed that TRPM2-AS was negatively correlated with miR-497-5p (Fig. 4G).

We analyzed the expression of miR-497-5p in UCEC of TCGA database and found that miR-497-5p was decreased in tumor tissues compared with normal group (Fig. 4H). We also analyzed miR-497-5p’s level of clinical samples and EC cell lines by qRT-PCR, and the results were consistent with TCGA databases (Fig. 4I, J). Our data demonstrates that TRPM2-AS binds with miR-497-5p and negatively correlated with miR-497-5p.

To explore miR-495-5p’s role in regulating the proliferation, invasion, migration and angiogenesis, we transfected miR-497-5p inhibitor, sh-TRPM2-AS or the combination of miR-497-5p inhibitor and sh-TRPM2-AS into Ishikawa or AN3CA cells. After 48 h, we conducted the EdU staining, transwell and in vitro HUVEC tube formation. EdU staining results revealed that inhibition of miR-495-5p promoted the proliferation of EC cells. However, knockdown of TRMP2-AS has the opposite effect and inhibition of miR-497-5p reversed the effect of sh-TRMP2-AS, which indicates that TRMP2-AS regulates the proliferation of EC cells through miR-497-5p (Fig. S4A). The miR-497-5p inhibitor promoted the invasion and migration of the Ishikawa and AN3CA cells. Whereas, sh-TRMP2-AS inhibit the invasion and migration of ECs, which would be reversed by inhibition of miR-497-5p (Fig.S4B-C). HUVEC tube formation assay showed that conditional medium of miR-497-5p inhibitor transfected EC cells promoted the tube formation of HUVECs compared with control group. However, sh-TRMP2-AS group has the opposite effect, and the trend could be reversed by the combination of miR-497-5p inhibitor and sh-TRMP2-AS (Fig. S4D).

### SPP1 promotes EC progression and angiogenesis

Based on the ceRNA network, we predicted the potential binding sites of miR-497-5p and SPP1 (Fig. 4K). Luciferase assay showed that miR-497-5p decreased the activity of SPP1 reporter in Ishikawa and AN3CA cells, and mutation of the biding site abolished this effect (Fig. 4L). Pearson curve analysis also showed that miR-497-5p was negatively correlated with SPP1 in EC clinical samples (Fig. 4M). The qRT-PCR and WB results reveal that after the transfection of miR-497-5p mimic into Ishikawa and AN3CA cells, both the RNA and protein level of SPP1 were decreased. miR-497-5p inhibitor group has the opposite trend (Fig.S5A-B). These results proved that SPP1 is a direct target gene of miR-497-5p.

**Fig. 5:**
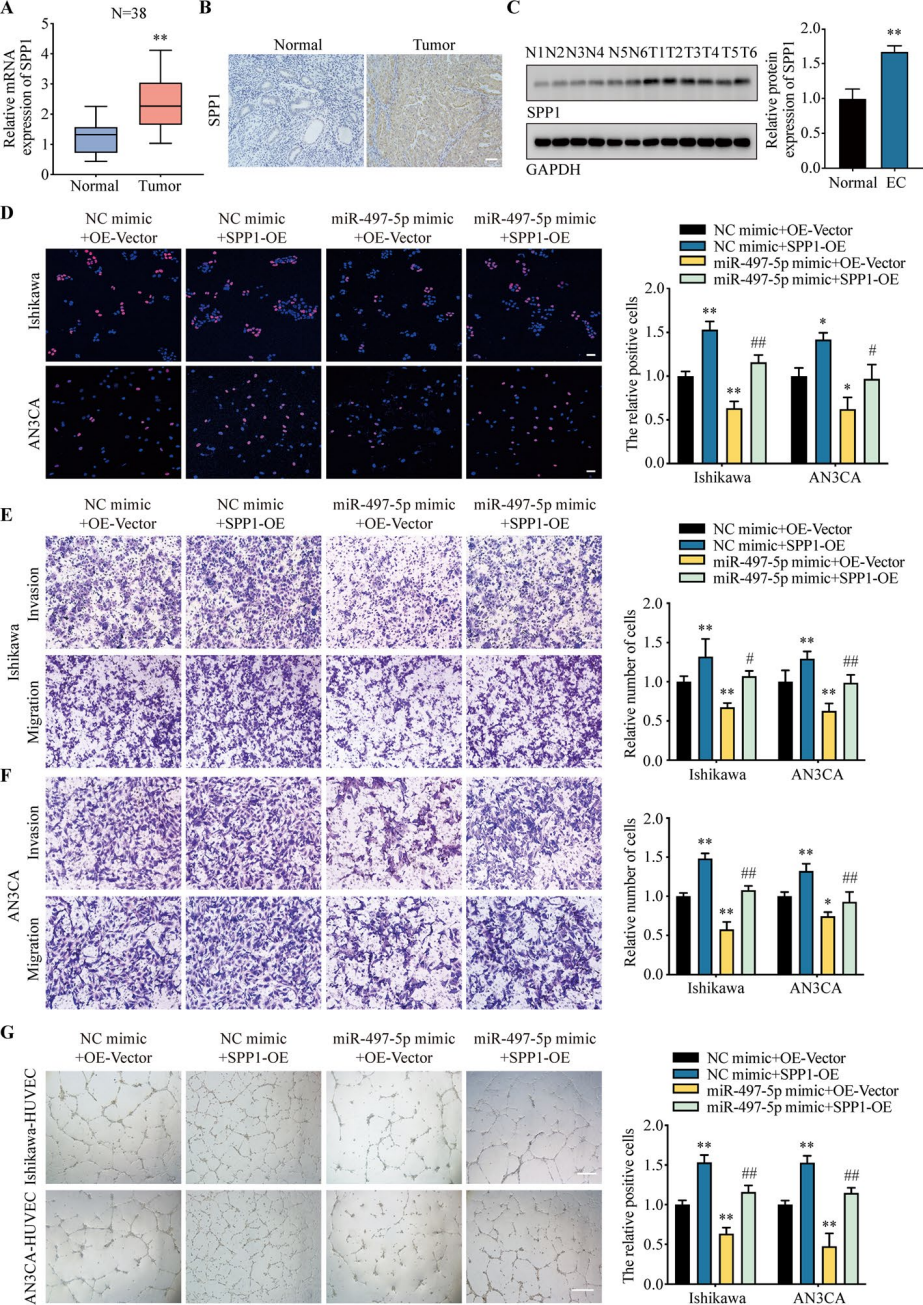
MiR-495-5p regulates the proliferation, invasion and migration of EC cells through SPP1. **A** The mRNA level of SPP1 in EC samples. **B** The expression of SPP1 in clinical specimens were detected by IHC. **C** The protein level of SPP1 in clinical samples were analyzed by WB. **D** After co-transfection of different combinations of miR-495-5p mimic, SPP1-OE or control vectors, the proliferation ability of Ishikawa or AN3CA cells were detected by EdU. **E**, **F** The invasion and migration ability of the vectors transfected Ishikawa or AN3CA cells were detected by transwell assay. **G** HUVEC tube formation assay revealed the effects of miR-495-5p on angiogenesis. Data were representative of three independent experiments and values were expressed in mean ± SD. (One-way ANOVA or Student’s *t*-test; *P < 0.05, **P < 0.01 as compared with normal or NC mimic + OE-Vector; # *P* < 0.05, ## *P* < 0.01 as compared with miR-497-5p mimic + OE-Vector)

We also analyzed the expression level of SPP1 in TCGA database and clinical samples. According to TCGA database, SPP1 were significantly upregulated in many tumors, including UCEC, breast invasive carcinoma (BRCA), lung adenocarcinoma (LUAD) and colon adenocarcinoma (COAD) (Fig. S5C). We used qRT-PCR, immunohistochemistry (IHC) and WB to detect the expression of SPP1 in clinical samples. The results were consistent with the TCGA database. Compared with normal tissues, SPP1 is highly expressed in EC tissues (Fig. 5A–C). SPP1 was also upregulated in EC cell lines (Fig. S5D-E).

To test the knockdown and overexpression efficiency of sh-SPP1 and SPP1-OE lentivirus in EC cells, qRT-PCR and WB assays were performed. Compared with the control group, the expression level of SPP1 in knockdown and overexpression groups were significantly changed after the infection of lentivirus (Fig. S5F-G). EdU staining showed that overexpression of SPP1 significantly promoted the proliferation of EC cells compared with control group. While miR-497-5p mimic has the opposite effect and overexpression of SPP1 reversed miR-497-5p’s effect (Fig. 5D). Transwell assay revealed that SPP1 promoted the invasion and migration of EC cells, miR-497-5p mimic decreased the migration of EC cells and this effect could be reversed by SPP1 (Fig. 5E, F). HUVEC in vitro tube formation assay also showed that SPP1 promoted the angiogenesis, and miR-497-5p inhibited the angiogenesis (Fig. 5G). The above results indicate that miR-497-5p inhibited the proliferation, invasion, migration and angiogenesis of EC cells through sponging SPP1.

In order to figure out whether TRPM2-AS regulates the expression of SPP1, we transfected sh-TRPM2-AS-2, sh-TRPM2-AS-3 and TRPM2-AS-OE plasmid into Ishikawa and AN3CA cells. Compared with the negative control group, the mRNA and protein level of SPP1 were reduced in sh-TRPM2-AS group and increased in the TRPM2-AS overexpression group (Fig. S5H-I).

### TRPM2-AS regulates angiogenesis by promoting the polarization of M2 macrophages

**Fig. 6:**
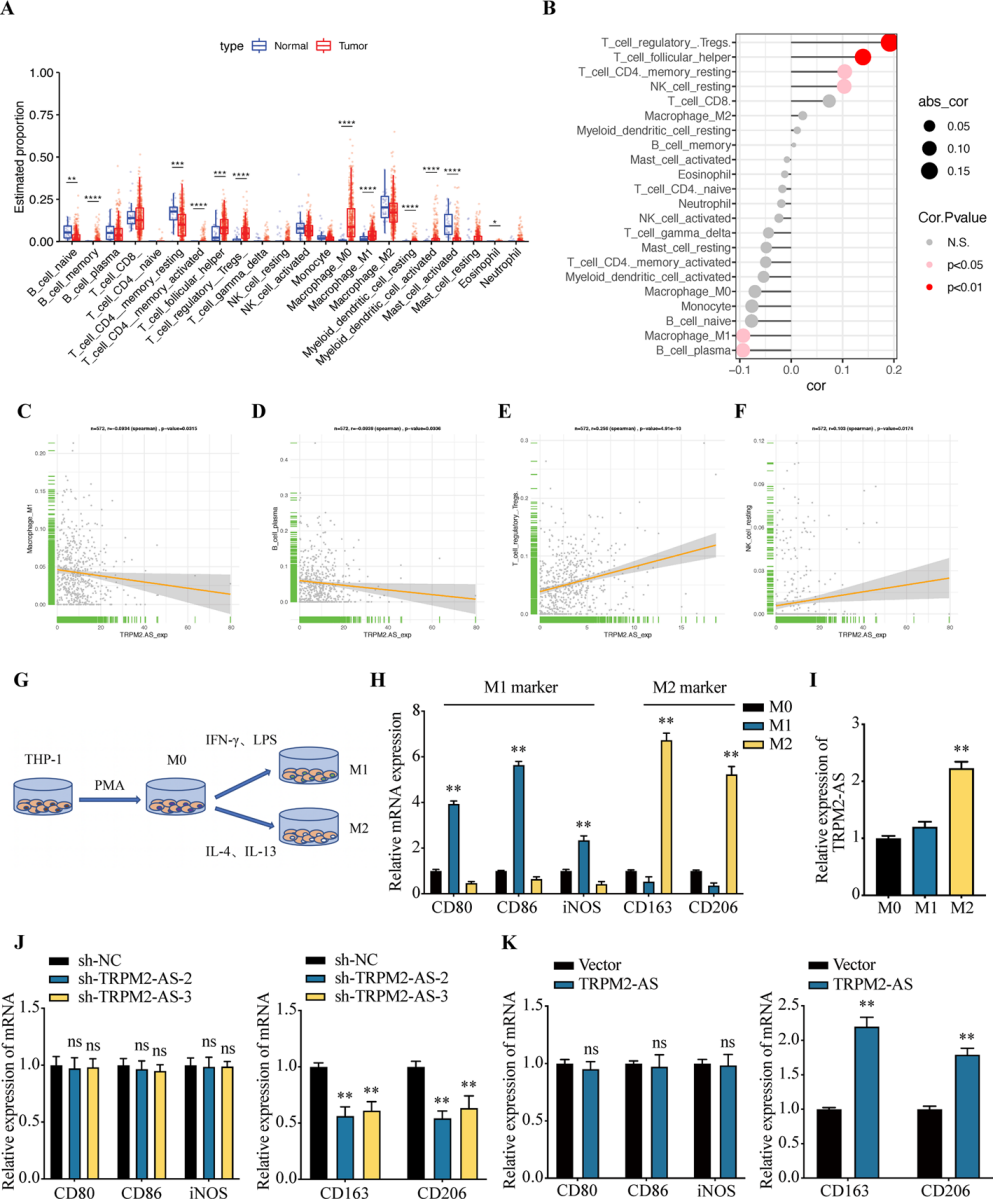
Analysis of the correlation between TRPM2-AS and immune cell infiltration in EC. **A** The infiltration abundance of 22 types of immune cells in EC. **B** The correlation between TRPM2-AS and 22 types of immune cell infiltration in EC. **C** TRPM2-AS was positively correlated with the infiltration of regulatory T cells. **D** TRPM2-AS was positively correlated with the infiltration of NK cells. **E** TRPM2-AS was negatively correlated with the infiltration of M1 macrophages. **F** TRPM2-AS was negatively correlated with the infiltration plasma B cells. **G** Schematic diagram shows the differentiation and polarization of M1 and M2 macrophages from THP-1 cells. **H** mRNA expression of M1 and M2 macrophage markers were detected by qRT-PCR. **I** Detection of TRPM2-AS in M0, M1and M2 macrophages. The expression of TRPM2-AS was significantly increased in M2 macrophages. **J** The mRNA expression of M1 and M2 macrophage markers were studied by qRT-PCR after knockdown of TRPM2-AS. **K** The mRNA expression of M1 and M2 macrophage markers were examined by qRT-PCR after overexpression of TRPM2-AS. Data were representative of three independent experiments and values were expressed in mean ± SD. (One-way ANOVA or Student’s *t*-test; *P < 0.05, **P < 0.01; # *P* < 0.05, ## *P* < 0.01)

To investigate the infiltration abundance of immune cells in EC, we conducted the CIBERSORT analysis. The results showed that the infiltration levels of 12 immune cell subtypes shows statistical difference between EC tumor tissues and normal tissues (Fig. 6A). The infiltration of naive B cell, resting memory CD4 T cells and activated mast cells were decreased in EC tissues, but the infiltration of memory B cell, activated memory CD4 T cells, T follicular helper cells, regulatory T cells, M0 macrophage, M1 macrophage, resting myeloid dendritic cells, activated myeloid dendritic cells and eosinophil were increased in tumor tissues of EC. Next, we analyzed the spearman correlation between TRPM2-AS and 22 types of immune cells in EC tissues. As shown in Fig. 6B, TRPM2-AS was correlated with 6 types of immune cell infiltration (regulatory T cell, T follicular helper cells, resting memory CD4 T cells, and NK cells, M1macrophage, plasma B cells). TRPM2-AS was positively correlated with the infiltration of regulatory T cells (Fig. 6C, *r* = 0.256) and NK cells (Fig. 6D , *r* = 0.103), and was negatively correlated with M1 macrophage (Fig. 6E, *r* = − 0.0934) and plasma B cells (Fig. 6F, *r* = − 0.0939). These results indicated that TRPM2-AS plays a critical role in regulating the tumor immune microenvironment of EC.

Studies have shown that macrophage infiltration modulates angiogenesis [24, 25]. Bioinformatics analysis showed that TRPM2-AS was correlated with macrophages in EC. To test the role of TRPM2-AS in regulating the polarization of macrophages, we induced the THP-1 cells to differentiated into M0 macrophages by the exposure of PMA. Then M0 macrophages were incubated with IFN-γ and LPS to obtain M1 macrophages or with IL-4 and IL-13 to induce M2 polarization (Fig. 6G). The qRT-PCR results showed that CD80, CD86 and iNOS (M1 macrophage marker) were highly expressed in M1 macrophages, and CD163 and CD206 (M2 macrophage marker) were significantly increased in M2 macrophages compared with M0 macrophages. These indicate the successful differentiation of M1 and M2 macrophages (Fig. 6H). Compared with M0 macrophages, the expression of TRPM2-AS in M2 macrophages was increased (Fig. 6I), indicating that TRPM2-AS may play a role in the macrophage polarization. After PMA incubation for 24 h, THP-1 cells were transfected with TRPM2-AS. IFN-γ and LPS were added to induce M0 macrophages differentiate into M1 macrophages. IL-4 and IL-13 were used to induced M0 macrophages differentiate into M2 macrophages, then the expression level of macrophage markers was detected. After knockdown of TRPM2-AS, the expression of CD80, CD86 and iNOS did not change significantly, while the expression of M2 macrophage markers (CD163 and CD206) significantly decreased (Fig. 6J). After overexpression of TRPM2-AS, there was no significant change in the expression of M1 macrophage markers, but the expression of CD163 and CD206 were significantly increased (Fig. 6K). These results indicated that TRPM2-AS regulates the polarization of M2 macrophages.

**Fig. 7:**
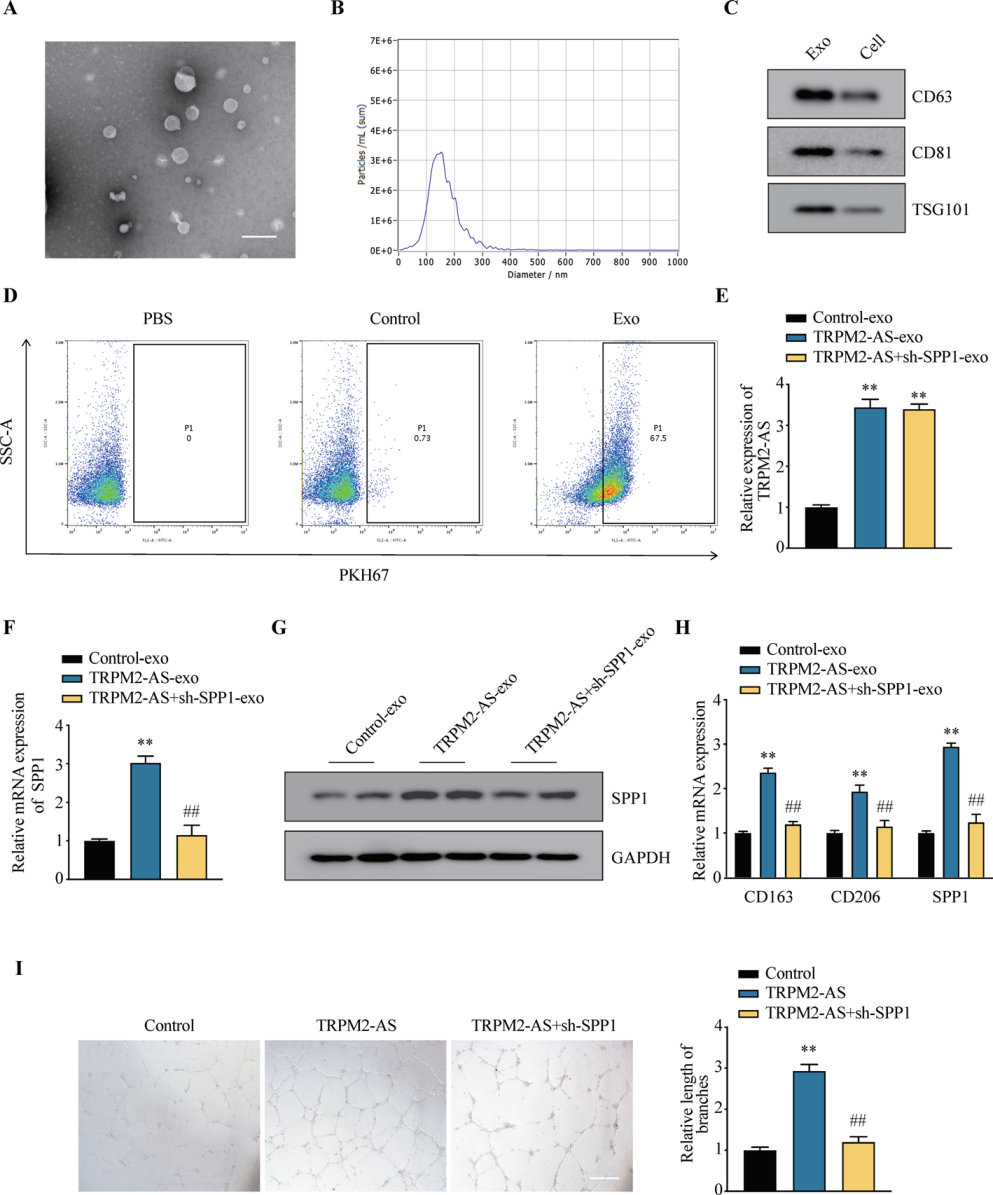
TRPM2-AS promotes the polarization of M2 macrophages and angiogenesis by up-regulating exosome derived SPP1. **A** Exosome morphology was observed by TEM. **B** NTA was used to detect the size and concentration of exosomes. **C** Expression of the biomarkers of exosomes were measured by WB. **D** The flow cytometry analysis revealed that PKH67 labeled exosomes were uptake by Ishikawa cells. **E** The mRNA expression of TRPM2-AS was measured by qRT-PCR. **F**, **G** The expression of SPP1 were analyzed by qRT-PCR (**F**) and WB (**G**). **H** The expression of M2 macrophage markers and SPP1 were analyzed by qRT-PCR. **I** Tube formation assay showed the angiogenesis ability of HUVECs after co-cultured with exosomes and macrophages. Data were representative of three independent experiments and values were expressed in mean ± SD. (One-way ANOVA or Student’s *t*-test; *P < 0.05, **P < 0.01)

Exosomes transmit information between cells, altering the phenotype of recipient cells in the TME [26]. To explore whether EC cells regulate macrophage polarization through secreting exosomes, we isolated exosomes from the cell culture medium of Ishikawa cells by supercentrifugation. Firstly, the extracted exosomes of morphology, particle size and markers were identified. The transmission electron microscopy (TEM) results showed that the exosomes were in circular or elliptical shape with intact membrane (Fig. 7A). Nanoparticle tracking analysis (NTA) revealed that the average diameter of the exosomes was 151.7 nm, and the concentration was 7.2E + 10 particles/mL (Fig. 7B). WB assay indicated that the samples were positive for surface markers of exosome including CD63, CD81 and TSG101 (Fig. 7C). The above results suggested that we successfully extracted exosomes derived from EC cells. Then, in order to examined the endocytosis rate of exosomes by macrophages, we stained the exosomes with the cell membrane labeling dye PKH67 and then co-cultured them with M0 macrophages for 24 h. Flow cytometry results revealed that M0 macrophages internalized the PKH67 labeled exosomes at the rate of 67.5% (Fig. 7D).

Subsequently, to explore whether TRPM2-AS regulates the macrophages polarization through SPP1, we infected Ishikawa cells with TRPM2-AS-OE or the combination of TRPM2-AS-OE and sh-SPP1 virus, and the exosomes were extracted from the cell culture medium. The qRT-PCR results revealed that the expression of TRPM2-AS in exosomes were significantly increased in TRPM2-AS-exo group compared with the control-exo group (Fig. 7E). The qRT-PCR and WB results showed that the expression of SPP1 in exosomes was significantly increased after the overexpression of TRPM2-AS and reduced in TRPM2-AS + sh-SPP1-exo group (Fig. 7F, G). To test whether TRPM2-AS induce the polarization of macrophages by SPP1, unpolarized M0 macrophages were co-cultured with control, TRPM2-AS, or TRPM2-AS + sh-SPP1 exosomes, and M2 macrophage markers were detected 48 h after the addition of IL-4 and IL-13. As shown in Fig. 7H, compared with the control group, the expression levels of M2 macrophage markers (CD163, CD206) were significantly increased in the TRPM2-AS-exo group and the trend was reversed by the knockdown of SPP1. To investigate whether the TRPM2-AS promote angiogenesis by SPP1, we co-cultured HUVECs with the conditioned medium of macrophages, and then conducted the tube formation assay. The results suggested that the conditioned medium of macrophages overexpressing TRPM2-AS promote angiogenesis of HUVECs, while knockdown of SPP1 reversed the promoting effect of TRPM2-AS on angiogenesis of HUVECs (Fig. 7I). Taken together, our results provide compelling evidence that EC cells induced the polarization of M2 macrophages and promoted angiogenesis of HUVECs by secreting SPP1 enriched exosomes.

### TRPM2-AS promotes EC tumor formation in nude mice

**Fig. 8:**
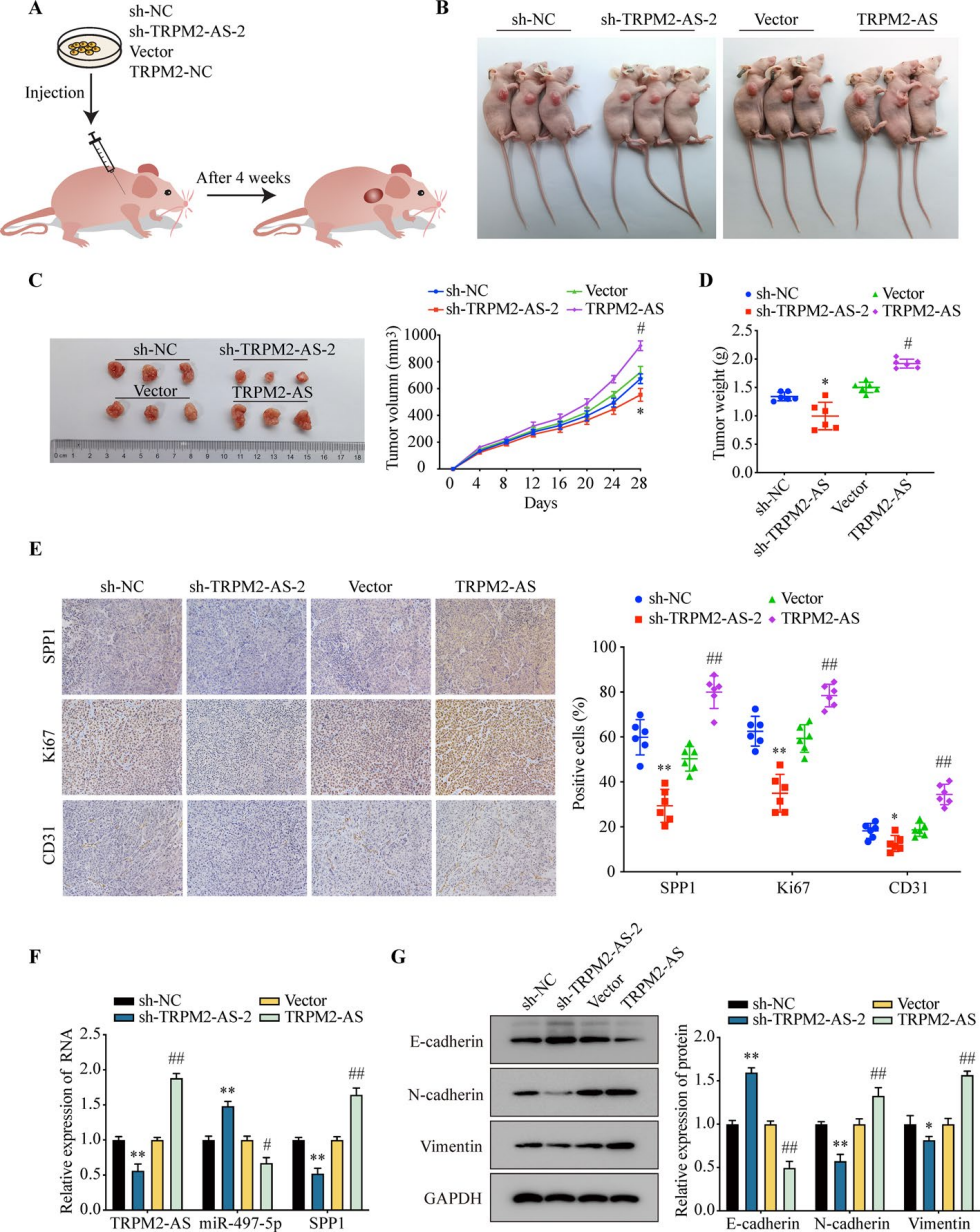
TRPM2-AS promotes EC tumor formation in nude mice. **A** The diagram of model for subcutaneous tumor formation in nude mice. **B** Mice injected with Ishikawa cells that stably expressing TRPM2-AS, sh-TRPM2-AS or the control vectors at the quantities of 1 × 10^6^. **C** Tumors dissected from the nude mice. **D** Tumor growth curves showed the tumor volume after the implantation of EC cells. **E** Average tumor weight in each group. **F** IHC staining of SPP1, Ki67 and CD31. **G** The mRNA expression of TRPM2-AS, miR-497-5p and SPP1 were detected by qRT-PCR. **H** EMT markers’ expression level were measured by WB. Data were representative of three independent experiments and values were expressed in mean ± SD. (One-way ANOVA or Student’s *t*-test; *P < 0.05, **P < 0.01; # *P* < 0.05, ## *P* < 0.01)

To explore the function of TRMP2-AS in vivo, we constructed the xenograft mouse model by subcutaneous injection the Ishikawa cells that stably expressed the sh-TRPM2-AS, sh-NC, TRPM2-AS-OE or control vector into nude mice (Fig. 8A).

As shown in Fig. 8B, Ishikawa cells successfully formed tumors in nude mice after subcutaneous injection. Compared with the control group, the transplanted tumors volume of the sh-TRPM2-AS group were significantly smaller and overexpression of TRPM2-AS significantly increased the transplanted tumors volume (Fig. 8C). The tumor weight of sh-TRPM2-AS group was significantly lower than the control group and overexpression of TRPM2-AS has the opposite effect (Fig. 8D).

The expression of SPP1, Ki67 (tumor proliferation marker) and CD31 were detected by IHC staining. The results showed that knockdown of TRPM2-AS significantly inhibited cell proliferation and angiogenesis in tumors. In contrast, overexpression of TRPM2-AS induced the upregulation of Ki67 and CD31 compared with control group (Fig. 8E). The qRT-PCR results also suggested that overexpression of TRPM2-AS inhibited miR-497-5p and promoted the expression of SPP1 in tumor of nude mice (Fig. 8F). WB results indicated that TRPM2-AS promoted EMT (Fig. 8G).

### Schematic diagram for the mechanisms of TRPM2-AS/miR-497-5p/SPP1 axis in EC

**Fig. 9:**
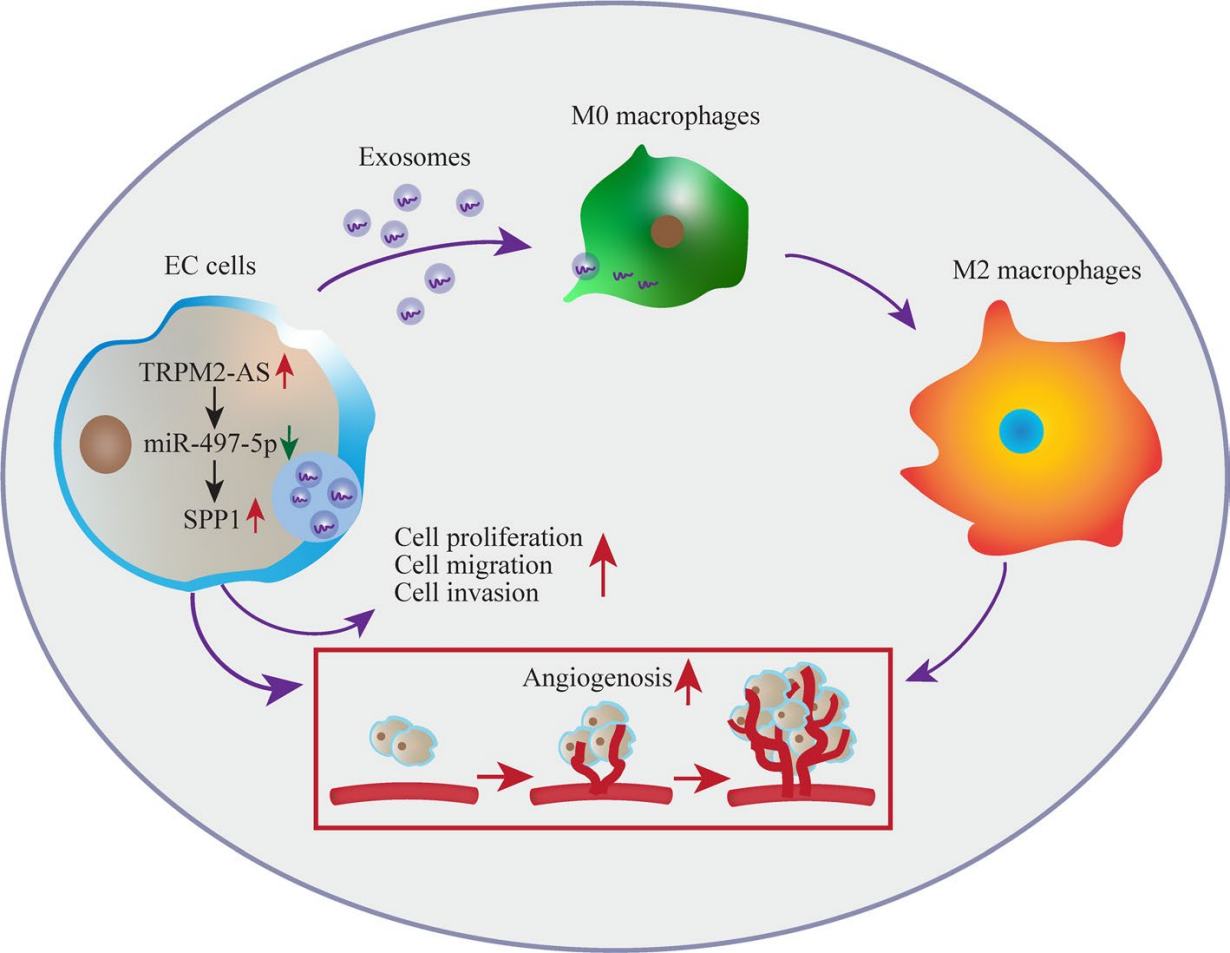
Schematic diagram. Schematic diagram for the mechanisms of TRPM2-AS/miR-497-5p/SPP1 axis in EC

In conclusion, we presented a model in which TRPM2-AS is abnormally highly expressed in EC and also a biomarker of poor prognosis. TRPM2-AS promotes the proliferation, invasion, migration of EC cells and angiogenesis of HUVECs through the miR-497-5p/SPP1 axis. On the other hand, TRPM2-AS increases the secretion of SPP1 enriched exosomes, which induces the polarization of M2 macrophages and promote angiogenesis (Fig. 9).

## Discussion

The therapeutic efficacy of advanced and recurrent EC is still limited despite extensive research in this area. Angiogenesis plays crucial roles in the development of EC, and the concept of treating malignant tumors by inhibiting angiogenesis was first proposed by Judah Folkman in 1971 [27]. Neovascularization stimulates the expression of a variety of growth factors, which directly promotes the growth of adjacent tumor cells. Angiogenesis is a prerequisite for tumor development and metastasis [28], since it provides nutrition and oxygen for tumor cells. Recently, targeted therapies of angiogenesis in EC have shown great advantages. In 2019, the FDA approved lenvatinib, an oral polytyrosine kinase inhibitor that targets vascular endothelial growth factor receptor (VEGFR), fibroblast growth factor receptor (FGFR), platelet-derived growth factor receptor (PDGFR), and stem cell factor receptor (KIT) for the treatment of advanced or recurrent EC. However, about two-thirds of patients have grade 3–4 adverse reactions, which leads to the reduction of dose and termination of treatment [29]. Although anti-angiogenic drugs delay the progression of EC, the efficacy is still limited. The drug resistance and adverse drug reactions remain challenging for EC treatment.

LncRNA play an important role in regulating angiogenesis [30]. Studies have shown that lncRNA NR2F1-AS1 increased the expression of insulin-like growth factor-1 (IGF-1) through sponge absorption of miRNA-338-3p, and the activation of IGF-1 promoted the angiogenesis of HUVECs in breast cancer [31]. AFAP1-AS1 regulated the expression of VEGF through the absorbtion of miR545-3p, thus affecting the angiogenesis and invasion of EC [32]. In this study, we investigated the effect of TRPM2-AS on tumor angiogenesis and prognosis in EC. TRPM2-AS was positively correlated with histological grade, FIGO stage, lymph node, distant metastasis and poor prognosis of patients with EC. Our study shows that TRPM2-AS is a tumorigenic lncRNA that can promote the proliferation, invasion, migration and angiogenesis of EC.

Studies have revealed that the expression of miR-497-5p was decreased in EC tissues. Compared with endometrioid carcinomas, miR-497-5p expression is significantly reduced in non-endometrioid uterine carcinomas such as papillary serous carcinomas, clear cell tumors and carcinosarcomas. Besides, decreased expression of miR-497-5p is positively correlated with histological grade, FIGO stage, lymph node and distant metastasis of EC, suggesting that miR-497-5p is correlated with the occurrence and progression of EC [33]. Our study found that miR-497-5p expression is reduced in the cell lines and tissues of EC, overexpression of miR-497-5p inhibited the proliferation, invasion, migration and angiogenesis of EC in vitro. Studies have shown that lncRNAs influence tumor development and regulate mRNA expression through absorbing miRNA [34]. In situ hybridization analysis showed that TRPM2-AS was mainly localized in the cytoplasm of EC cells. As predicted by ceRNA network, dual luciferase reporter assay showed that TRPM2-AS could act as a competitive ceRNA for miR-497-5p. Moreover, TRPM2-AS promoted the progression of EC by upregulating SPP1.

SPP1 plays important roles in regulating tumor growth, EMT, metastasis and angiogenesis [35]. In endothelial cells, SPP1 stimulates angiogenesis and regulates VEGF through phosphorylation and activation of PI3K/AKT and ERK1/2 pathways [36]. SPP1 is also significantly associated with adverse survival outcomes in various cancers [35]. SPP1 promotes the chemotherapy resistance of leukemia and solid tumors. Therefore, SPP1 is emerging as a novel target for the treatment of leukemia and solid tumors [37]. Our study also reveals that SPP1 is highly expressed in tumor tissues and cell lines of EC, and overexpression of SPP1 significantly promote angiogenesis in EC.

A higher tumor-associated macrophages (TAMs) infiltration level and M1/M2 ratio are associated with the formation of blood vessels and poor prognosis in most tumors [38–43]. M2 macrophages are the major component of tumor-infiltrating immune cells and they are closely related to tumor angiogenesis [44]. Therefore, targeting tumor associated M2 macrophages is a promising anti-tumor strategy. LncRNA can regulate the M1/M2 macrophages polarization, and regulating the secretion of pro-inflammatory or anti-inflammatory cytokines [45]. To explore the function of TRPM2-AS in EC, we used CIBERSORT database to analyze the level of immune cell infiltration in the TME. The results show that TRPM2-AS is associated with TAMs, and TAMs play critical roles in angiogenesis and tumor progression. Results show that overexpression of TRPM2-AS promotes the polarization of macrophages from M0 to M2. Study have shown that SPP1 is a chemokine of macrophage, which can maintain the M2 macrophages, and blockade of SPP1 impair the macrophages recruitment ability of tumors [46]. Therefore, we hypothesized that TRPM2-AS induced the polarization of M2 macrophages by promoting the secretion of SPP1 enriched exosomes. The results showed that EC cells derived exosomes which enriched of SPP1 could be endocytosed by macrophages and induced the polarization of M2 macrophages, which promoted the angiogenesis of HUVECs.

Understanding of the molecular mechanisms underlying the progression of EC are crucial for the treatment. In this study, we found that TRPM2-AS worked as a tumorigenic lncRNA in EC, and promoting the M2 macrophage polarization of EC through miR-497-5p/SPP1 axis. EC cells regulated the macrophages polarization by secreting SPP1 enriched exosomes, which finally promoted the angiogenesis of endometrial cancer.

## Conclusions

Overall, TRPM2-AS is up-regulated and associated with poor prognosis in EC. We also found that TRPM2-AS promotes the proliferation, invasion, migration of EC cells and angiogenesis of HUVECs through the miR-497-5p/SPP1 axis. Moreover, EC cells secreted SPP1 enriched exosomes, which mediate the communication within TME, and induce the polarization of M2 macrophages to promote angiogenesis. Therefore, this study offers a better understanding of TRPM2-AS’s role in regulating EC angiogenesis and provides a novel target for the treatment of EC.

### Supplementary Information

Supplementary Material 1. Fig. 1 Clinical characteristics of 4 lncRNAs in EC. (A) The heat map showed the expression of 4 lncRNAs in high-risk and low-risk groups based on clinical characteristics. (B-C) Univariate (B) and Multivariate (C) Cox regression analysis of the association between clinicopathological factors and OS. (D) The predictive nomogram of EC patients.

Supplementary Material 2. Fig. 2 The predicted ceRNA network of angiogenesis related lncRNA in EC. (A) The ceRNA network of HAND2-AS1 and TRPM2-AS in EC.

Supplementary Material 3. Fig. 3 TRPM2-AS regulates the metastasis and angiogenesis of EC cells. (A-B) WB analysis of the expression of EMT markers and angiogenesis maker CD31 in EC cells after knock down and overexpression of TRPM2-AS in Ishikawa (A) and AN3CA (B) cells. (C-D) The mRNA expression of EMT markers and CD31 were analyzed by qRT-PCR in Ishikawa (C) and AN3CA (D) cells. Data were representative of three independent experiments and values were expressed in mean ± SD. (One-way ANOVA or Student’s t-test; *P < 0.05, **P < 0.01as compared with normal or sh-NC; # *P* < 0.05, ## *P* < 0.01 as compared with vector).

Supplementary Material 4. Fig. 4 Knockdown of TRPM2-AS inhibits the proliferation, invasion, migration and angiogenesis of EC cells through miR-497-5p. (A) The proliferative ability of Ishikawa and AN3CA cells that treated with different combinations of miR-497-5p inhibitor, sh-TRPM2-AS and the control vectors were analyzed by EdU staining. (B-C) Invasion and migration of Ishikawa (B) or AN3CA (C) cells in each group were detected by Transwell assay. (D) Tube formation assay shows the angiogenesis ability of HUVEC in each group. Data were representative of three independent experiments and values were expressed in mean ± SD. (One-way ANOVA or Student’s t-test; *P < 0.05, **P < 0.01as compared with normal or sh-NC + NC inhibitor; # *P* < 0.05, ## *P* < 0.01 as compared with sh-TRPM2-AS + NC inhibitor).

Supplementary Material 5. Fig. 5 TRPM2-AS regulates the expression of SPP1 by sponging miR-497-5p in EC cells. (A-B) The expression of SPP1 in miR-497-5p mimic or miR-497-5p inhibitor transfected EC cells were analyzed by qRT-PCR (A) and WB (B). (C) The expression of SPP1 in different cancers based on TCGA datasets. (D) The expression of SPP1 in EC cells were analyzed by WB. (E) The mRNA level of SPP1 in EC cells were measured by qRT-PCR. (F-G) The expression of SPP1 in sh-SPP1, sh-SPP2 or SPP1-OE groups were measured by qRT-PCR (F) and WB (G). (H-I) The expression of SPP1 in sh-TRPM2-AS-1, sh-TRPM2-AS-2, sh-TRPM2-AS-3 or TRPM2-AS transfected EC cells were analyzed by qRT-PCR (H) and WB (I). Data were representative of three independent experiments and values were expressed in mean ± SD. (One-way ANOVA or Student’s t-test; *P < 0.05, **P < 0.01; # *P* < 0.05, ## *P* < 0.01).

## Data Availability

The data supporting the results of this study are available from the corresponding author on reasonable request.

## Abbreviations

ceRNA: Competing endogenous RNA
EC: Endometrial carcinoma
EMT: Epithelial-mesenchymal transition
GSEA: Gene set enrichment analyses
HUVEC: Human umbilical vein endotheial cell
LASSO: Least absolute shrinkage and selection operator
LncRNA: Long non-coding RNA
OS: Overall survival
RIP: RNA immunoprecipitation
TAMs: Tumor-associated macrophages
TCGA: The Cancer Genome Atlas
TME: Tumor microenvironment
UCEC: Uterine corpus endometrial carcinoma
VEGF: Vascular endothelial growth factor

## References

[1] Siegel RL, Miller KD, Wagle NS, Jemal A (2023). Cancer statistics, 2023. CA Cancer J Clin.

[2] Islami F, Ward EM, Sung H, Cronin KA, Tangka FKL, Sherman RL (2021). Annual report to the nation on the status of cancer, part 1: National Cancer Statistics. J Natl Cancer Inst.

[3] Makker V, MacKay H, Ray-Coquard I, Levine DA, Westin SN, Aoki D (2021). Endometrial cancer. Nat Rev Dis Primers.

[4] Liu H, Wan J, Chu J (2019). Long non-coding RNAs and endometrial cancer. Biomed Pharmacother.

[5] Zhou B, Yang H, Yang C, Bao YL, Yang SM, Liu J (2021). Translation of noncoding RNAs and cancer. Cancer Lett.

[6] Xu C, Huang Q, Zhang C, Xu W, Xu G, Zhao X (2018). Long non-coding RNA TRPM2-AS as a potential biomarker for hepatocellular carcinoma. Ir J Med Sci.

[7] Ding Y, Tan X, Abasi A, Dai Y, Wu R, Zhang T (2021). LncRNA TRPM2-AS promotes ovarian cancer progression and cisplatin resistance by sponging miR-138-5p to release SDC3 mRNA. Aging (Albany NY).

[8] Tian Y, Guan Y, Su Y, Yang T, Yu H (2021). TRPM2-AS promotes bladder cancer by targeting miR-22-3p and regulating GINS2 mRNA expression. Onco Targets Ther.

[9] Hyder SM, Stancel GM (2000). Regulation of VEGF in the reproductive tract by sex-steroid hormones. Histol Histopathol.

[10] Berger AA, Dao F, Levine DA (2021). Angiogenesis in endometrial carcinoma: therapies and biomarkers, current options, and future perspectives. Gynecol Oncol.

[11] Lee YC, Lheureux S, Oza AM (2017). Treatment strategies for endometrial cancer: current practice and perspective. Curr Opin Obstet Gynecol.

[12] Belli C, Trapani D, Viale G, D'Amico P, Duso BA, Della Vigna P (2018). Targeting the microenvironment in solid tumors. Cancer Treat Rev.

[13] Anderson NM, Simon MC (2020). The tumor microenvironment. Curr Biol.

[14] Ganesh K, Massagué J (2021). Targeting metastatic cancer. Nat Med.

[15] Jiang X, Wang J, Deng X, Xiong F, Zhang S, Gong Z (2020). The role of microenvironment in tumor angiogenesis. J Exp Clin Cancer Res.

[16] Erin N, Grahovac J, Brozovic A, Efferth T (2020). Tumor microenvironment and epithelial mesenchymal transition as targets to overcome tumor multidrug resistance. Drug Resist Update.

[17] Butti R, Kumar TVS, Nimma R, Banerjee P, Kundu IG, Kundu GC (2021). Osteopontin signaling in shaping tumor microenvironment conducive to malignant progression. Adv Exp Med Biol.

[18] Kazakova E, Rakina M, Sudarskikh T, Iamshchikov P, Tarasova A, Tashireva L (2023). Angiogenesis regulators S100A4, SPARC and SPP1 correlate with macrophage infiltration and are prognostic biomarkers in colon and rectal cancers. Front Oncol.

[19] Xu C, Sun L, Jiang C, Zhou H, Gu L, Liu Y (2017). SPP1, analyzed by bioinformatics methods, promotes the metastasis in colorectal cancer by activating EMT pathway. Biomed Pharmacother.

[20] Sangaletti S, Tripodo C, Sandri S, Torselli I, Vitali C, Ratti C (2014). Osteopontin shapes immunosuppression in the metastatic niche. Cancer Res.

[21] Raja UM, Gopal G, Shirley S, Ramakrishnan AS, Rajkumar T (2017). Immunohistochemical expression and localization of cytokines/chemokines/growth factors in gastric cancer. Cytokine.

[22] Giopanou I, Lilis I, Papaleonidopoulos V, Agalioti T, Kanellakis NI, Spiropoulou N (2017). Tumor-derived osteopontin isoforms cooperate with TRP53 and CCL2 to promote lung metastasis. Oncoimmunology.

[23] Yang X, Wang CC, Lee WYW, Trovik J, Chung TKH, Kwong J (2018). Long non-coding RNA HAND2-AS1 inhibits invasion and metastasis in endometrioid endometrial carcinoma through inactivating neuromedin U. Cancer Lett.

[24] Mohapatra S, Pioppini C, Ozpolat B, Calin GA (2021). Non-coding RNAs regulation of macrophage polarization in cancer. Mol Cancer.

[25] Mantovani A, Allavena P, Marchesi F, Garlanda C (2022). Macrophages as tools and targets in cancer therapy. Nat Rev Drug Discov.

[26] Kourembanas S (2015). Exosomes: vehicles of intercellular signaling, biomarkers, and vectors of cell therapy. Annu Rev Physiol.

[27] Folkman J (1971). Tumor angiogenesis: therapeutic implications. N Engl J Med.

[28] Le Querrec A, Duval D, Tobelem G (1993). Tumour angiogenesis. Baillieres Clin Haematol.

[29] Makker V, Taylor MH, Aghajanian C, Oaknin A, Mier J, Cohn AL (2020). Lenvatinib plus pembrolizumab in patients with advanced endometrial cancer. J Clin Oncol.

[30] Zhao Z, Sun W, Guo Z, Zhang J, Yu H, Liu B (2020). Mechanisms of lncRNA/microRNA interactions in angiogenesis. Life Sci.

[31] Zhang Q, Li T, Wang Z, Kuang X, Shao N, Lin Y (2020). lncRNA NR2F1-AS1 promotes breast cancer angiogenesis through activating IGF-1/IGF-1R/ERK pathway. J Cell Mol Med.

[32] Zhong Y, Wang Y, Dang H, Wu X (2020). LncRNA AFAP1-AS1 contributes to the progression of endometrial carcinoma by regulating miR-545-3p/VEGFA pathway. Mol Cell Probes.

[33] Fridrichova I, Kalinkova L, Karhanek M, Smolkova B, Machalekova K, Wachsmannova L (2020). miR-497-5p decreased expression associated with high-risk endometrial cancer. Int J Mol Sci.

[34] Salmena L, Poliseno L, Tay Y, Kats L, Pandolfi PP (2011). A ceRNA hypothesis: the Rosetta Stone of a hidden RNA language?. Cell.

[35] Dong B, Wu C, Huang L, Qi Y (2021). Macrophage-related SPP1 as a potential biomarker for early lymph node metastasis in lung adenocarcinoma. Front Cell Dev Biol.

[36] Ramchandani D, Weber GF (2015). Interactions between osteopontin and vascular endothelial growth factor: implications for cancer. Biochim Biophys Acta.

[37] Mirzaei A, Mohammadi S, Ghaffari SH, Yaghmaie M, Vaezi M, Alimoghaddam K (2018). Osteopontin b and c splice isoforms in leukemias and solid tumors: angiogenesis alongside chemoresistance. Asian Pac J Cancer Prev.

[38] Cassetta L, Pollard JW (2018). Targeting macrophages: therapeutic approaches in cancer. Nat Rev Drug Discov.

[39] Cortese N, Carriero R, Laghi L, Mantovani A, Marchesi F (2020). Prognostic significance of tumor-associated macrophages: past, present and future. Semin Immunol.

[40] DeNardo DG, Ruffell B (2019). Macrophages as regulators of tumour immunity and immunotherapy. Nat Rev Immunol.

[41] Engblom C, Pfirschke C, Pittet MJ (2016). The role of myeloid cells in cancer therapies. Nat Rev Cancer.

[42] Locati M, Curtale G, Mantovani A (2020). Diversity, mechanisms, and significance of macrophage plasticity. Annu Rev Pathol.

[43] Mantovani A, Marchesi F, Malesci A, Laghi L, Allavena P (2017). Tumour-associated macrophages as treatment targets in oncology. Nat Rev Clin Oncol.

[44] Sammarco G, Gadaleta CD, Zuccalà V, Albayrak E, Patruno R, Milella P (2018). Tumor-associated macrophages and mast cells positive to tryptase are correlated with angiogenesis in surgically-treated gastric cancer patients. Int J Mol Sci.

[45] Ghafouri-Fard S, Abak A, Tavakkoli Avval S, Shoorei H, Taheri M, Samadian M (2021). The impact of non-coding RNAs on macrophage polarization. Biomed Pharmacother.

[46] Wei J, Marisetty A, Schrand B, Gabrusiewicz K, Hashimoto Y, Ott M (2019). Osteopontin mediates glioblastoma-associated macrophage infiltration and is a potential therapeutic target. J Clin Invest.

