# Supplementary figures and images for: LncRNA TRPM2-AS promotes endometrial carcinoma progression and angiogenesis via targeting miR-497-5p/SPP1 axis
Source: Cell Mol Biol Lett. 2024 Jul 2;29:93. doi: 10.1186/s11658-024-00612-7 (PMC11218065; doi:10.1186/s11658-024-00612-7)

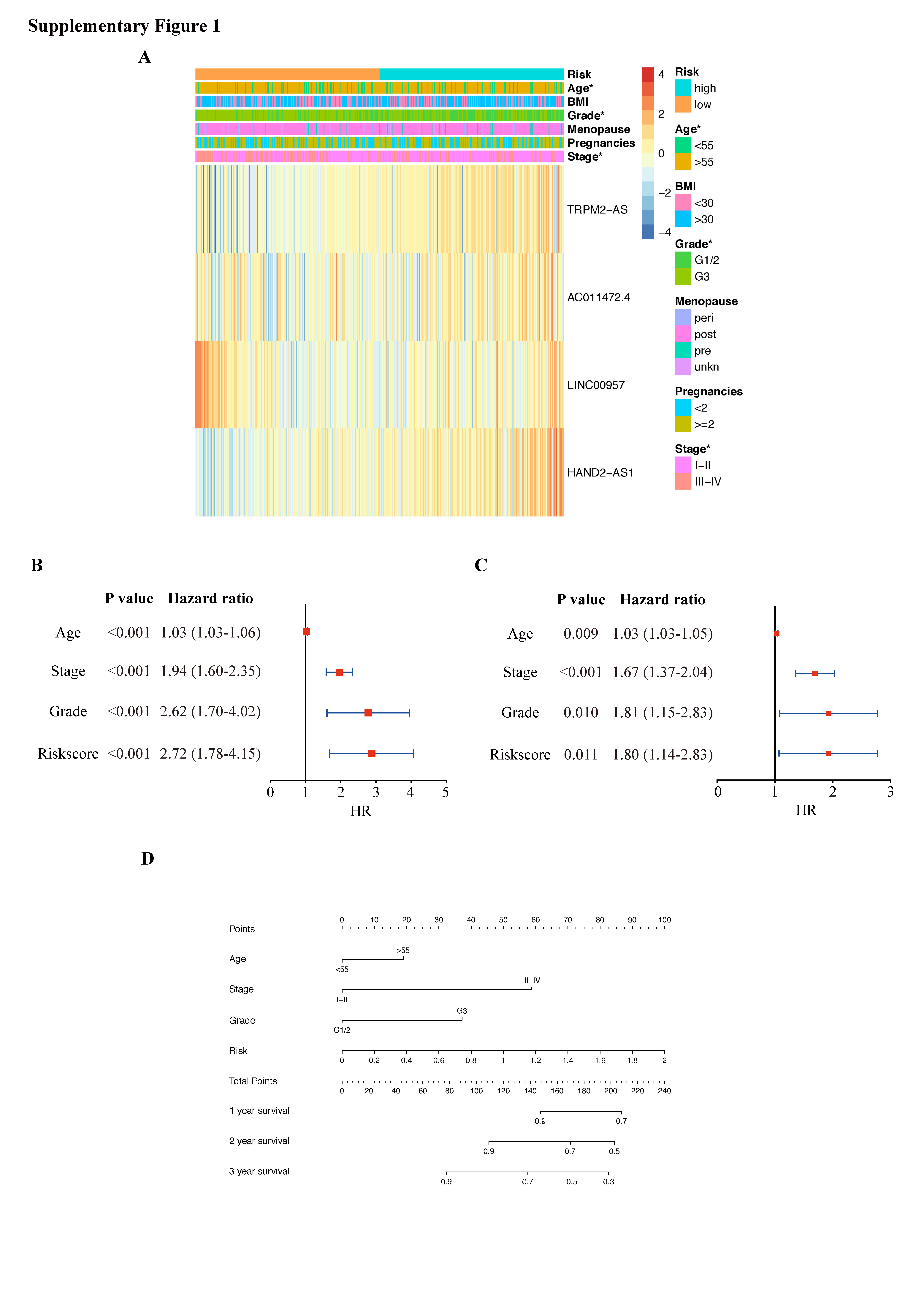

Supplement: Supplementary file 1 — Supplementary Material 1. Fig. 1 Clinical characteristics of 4 lncRNAs in EC. (A) The heat map showed the expression of 4 lncRNAs in high-risk and low-risk groups based on clinical characteristics. (B-C) Univariate (B) and Multivariate (C) Cox regression analysis of the association between clinicopathological factors and OS. (D) The predictive nomogram of EC patients. [file 11658_2024_612_MOESM1_ESM.tiff]

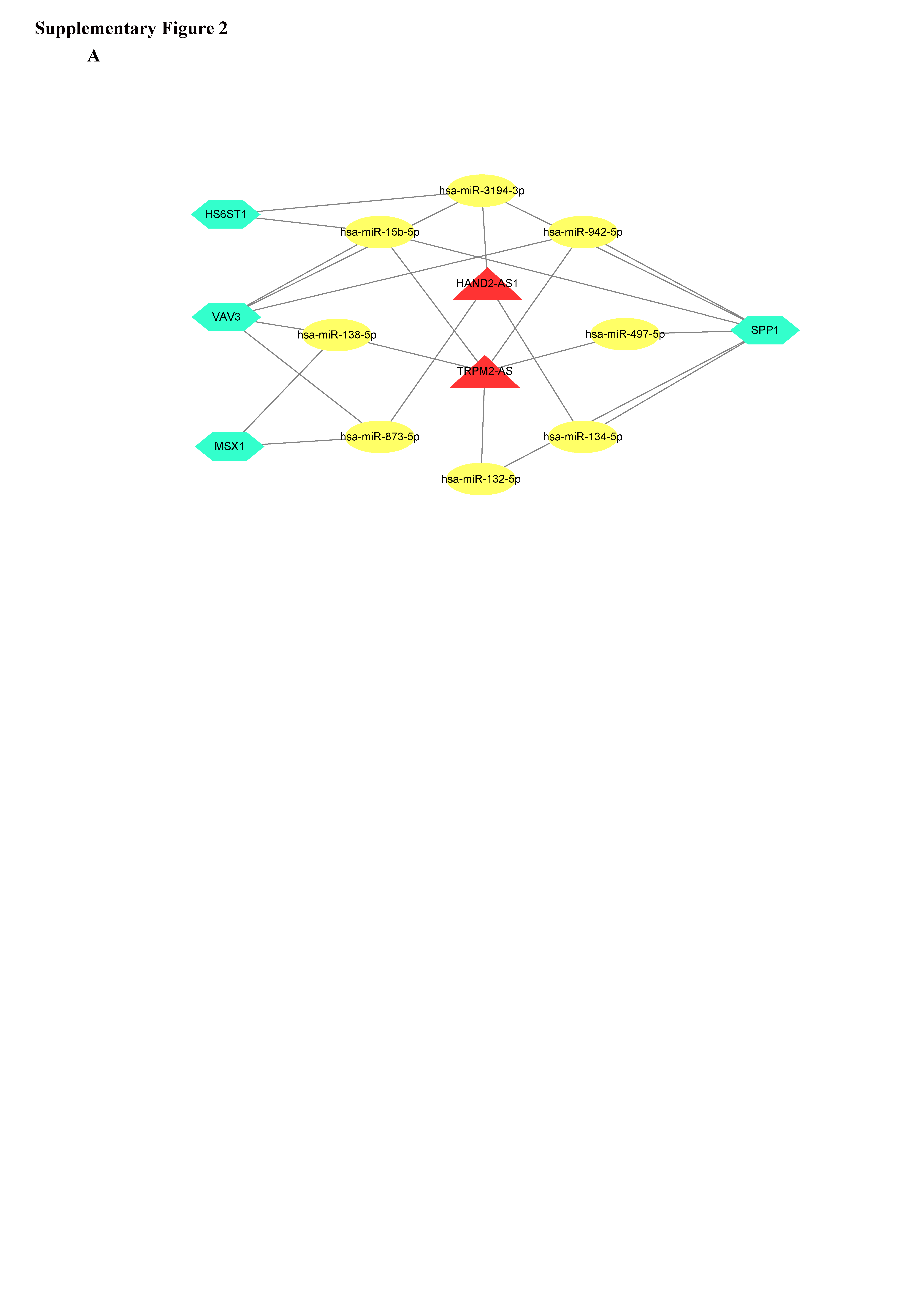

Supplement: Supplementary file 2 — Supplementary Material 2. Fig. 2 The predicted ceRNA network of angiogenesis related lncRNA in EC. (A) The ceRNA network of HAND2-AS1 and TRPM2-AS in EC. [file 11658_2024_612_MOESM2_ESM.tiff]

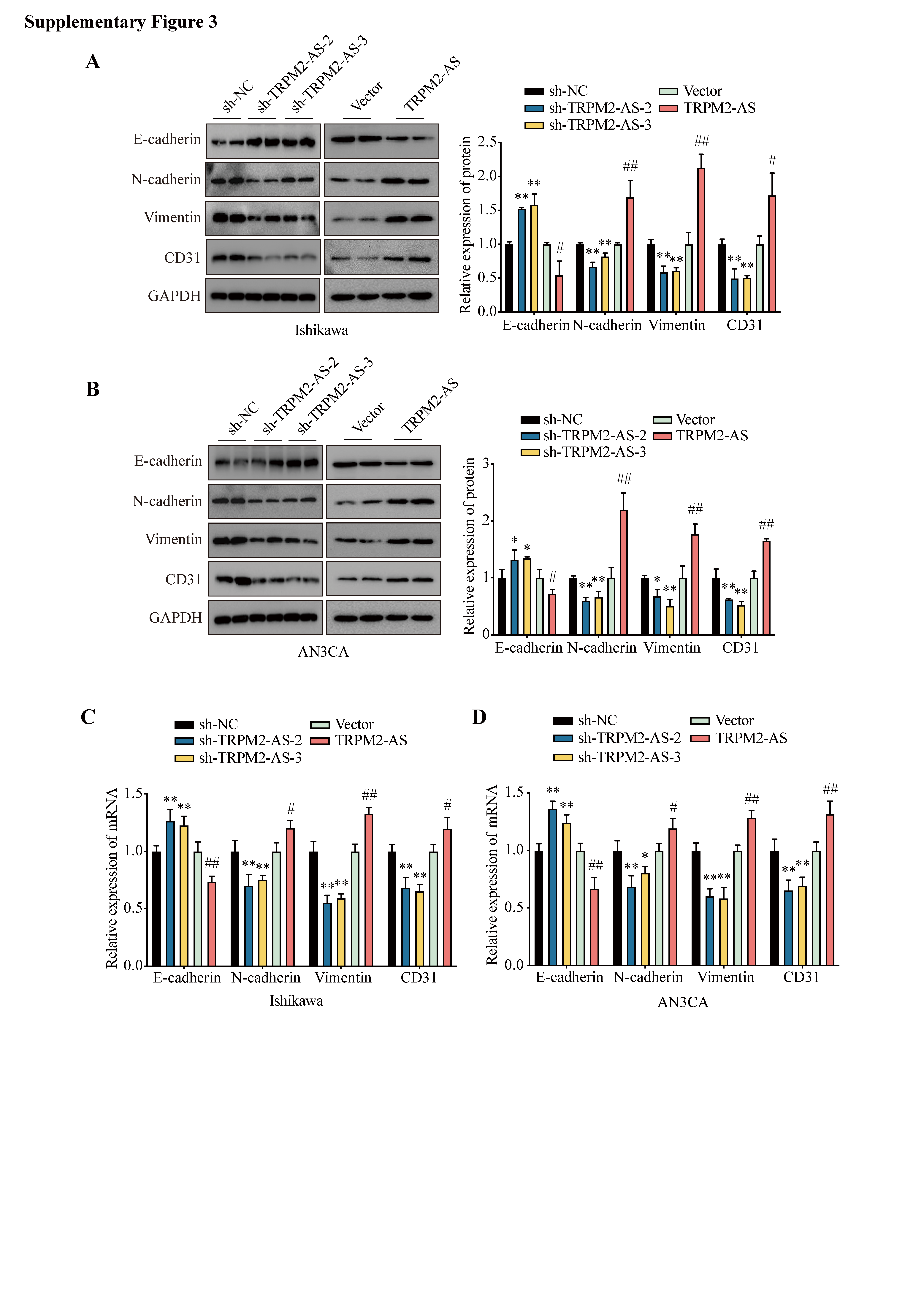

Supplement: Supplementary file 3 — Supplementary Material 3. Fig. 3 TRPM2-AS regulates the metastasis and angiogenesis of EC cells. (A-B) WB analysis of the expression of EMT markers and angiogenesis maker CD31 in EC cells after knock down and overexpression of TRPM2-AS in Ishikawa (A) and AN3CA (B) cells. (C-D) The mRNA expression of EMT markers and CD31 were analyzed by qRT-PCR in Ishikawa (C) and AN3CA (D) cells. Data were representative of three independent experiments and values were expressed in mean ± SD. (One-way ANOVA or Student’s t-test; *P < 0.05, **P < 0.01as compared with normal or sh-NC; # P < 0.05, ## P < 0.01 as compared with vector). [file 11658_2024_612_MOESM3_ESM.tiff]

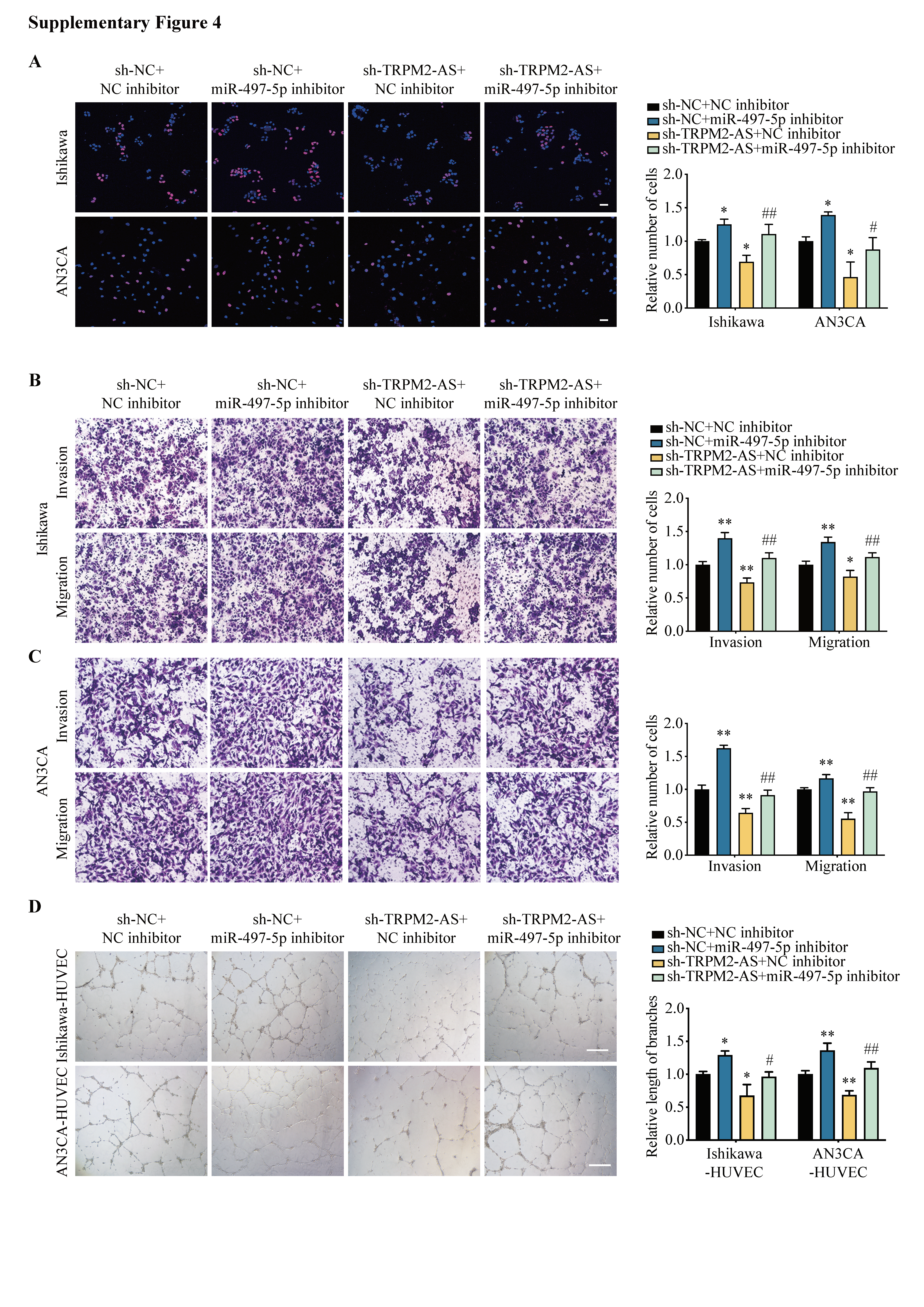

Supplement: Supplementary file 4 — Supplementary Material 4. Fig. 4 Knockdown of TRPM2-AS inhibits the proliferation, invasion, migration and angiogenesis of EC cells through miR-497-5p. (A) The proliferative ability of Ishikawa and AN3CA cells that treated with different combinations of miR-497-5p inhibitor, sh-TRPM2-AS and the control vectors were analyzed by EdU staining. (B-C) Invasion and migration of Ishikawa (B) or AN3CA (C) cells in each group were detected by Transwell assay. (D) Tube formation assay shows the angiogenesis ability of HUVEC in each group. Data were representative of three independent experiments and values were expressed in mean ± SD. (One-way ANOVA or Student’s t-test; *P < 0.05, **P < 0.01as compared with normal or sh-NC + NC inhibitor; # P < 0.05, ## P < 0.01 as compared with sh-TRPM2-AS + NC inhibitor). [file 11658_2024_612_MOESM4_ESM.tiff]

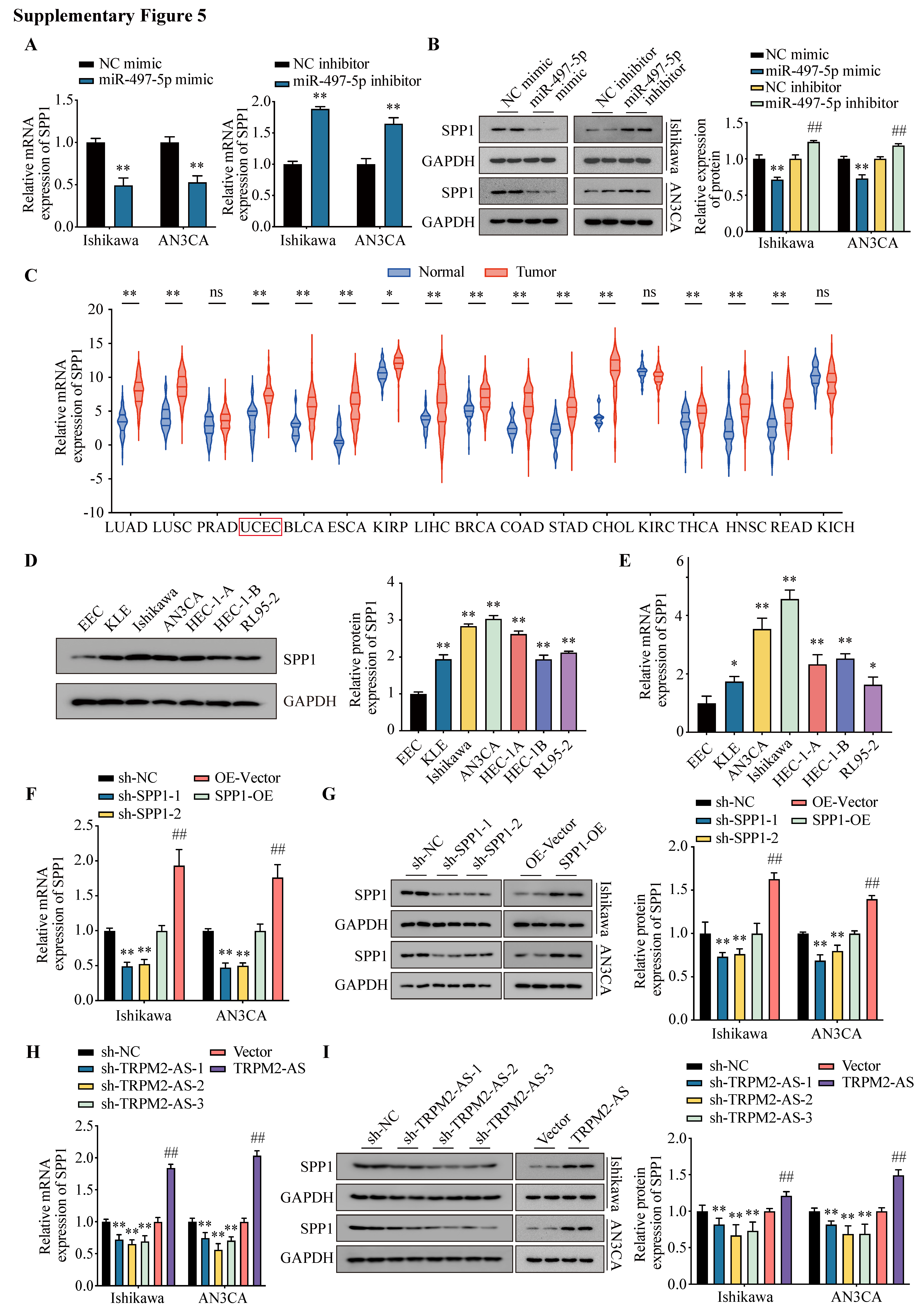

Supplement: Supplementary file 5 — Supplementary Material 5. Fig. 5 TRPM2-AS regulates the expression of SPP1 by sponging miR-497-5p in EC cells. (A-B) The expression of SPP1 in miR-497-5p mimic or miR-497-5p inhibitor transfected EC cells were analyzed by qRT-PCR (A) and WB (B). (C) The expression of SPP1 in different cancers based on TCGA datasets. (D) The expression of SPP1 in EC cells were analyzed by WB. (E) The mRNA level of SPP1 in EC cells were measured by qRT-PCR. (F-G) The expression of SPP1 in sh-SPP1, sh-SPP2 or SPP1-OE groups were measured by qRT-PCR (F) and WB (G). (H-I) The expression of SPP1 in sh-TRPM2-AS-1, sh-TRPM2-AS-2, sh-TRPM2-AS-3 or TRPM2-AS transfected EC cells were analyzed by qRT-PCR (H) and WB (I). Data were representative of three independent experiments and values were expressed in mean ± SD. (One-way ANOVA or Student’s t-test; *P < 0.05, **P < 0.01; # P < 0.05, ## P < 0.01). [file 11658_2024_612_MOESM5_ESM.tiff]
